# Crystal Structure and Magnetic Properties of Trinuclear Transition Metal Complexes (Mn^II^, Co^II^, Ni^II^ and Cu^II^) with Bridging Sulfonate-Functionalized 1,2,4-Triazole Derivatives

**DOI:** 10.3390/molecules26196020

**Published:** 2021-10-04

**Authors:** Andrea Moneo-Corcuera, Breogán Pato-Doldan, Irene Sánchez-Molina, David Nieto-Castro, José Ramón Galán-Mascarós

**Affiliations:** 1 Institute of Chemical Research of Catalonia (ICIQ), The Barcelona Institute of Science and Technology (BIST), Av. Països Catalans, 16, 43007 Tarragona, Spain; breogan.pato@gmail.com (B.P.-D.); isanchez@ICIQ.ES (I.S.-M.); dnieto@ICIQ.ES (D.N.-C.); 2 Departament de Química Física I Inorgànica, Universitat Rovira I Virgili, C/Marcel·lí Domingo, 43007 Tarragona, Spain; 3 ICREA, Passeig Lluís Companys 23, 08010 Barcelona, Spain

**Keywords:** linear trimers, crystal structure, magnetic exchange, coordination chemistry

## Abstract

Here we present the synthesis, structure and magnetic properties of complexes of general formula (Mn)(Me_2_NH_2_)_4_][Mn_3_(μ-L)_6_(H_2_O)_6_] and (Me_2_NH_2_)_6_[M_3_(μ-L)_6_(H_2_O)_6_] (M = Co^II^, Ni^II^ and Cu^II^); L^−2^ = 4-(1,2,4-triazol-4-yl) ethanedisulfonate). The trinuclear polyanions were isolated as dimethylammonium salts, and their crystal structures determined by single crystal and powder X-ray diffraction data. The polyanionic part of these salts have the same molecular structure, which consists of a linear array of metal(II) ions linked by triple N1-N2-triazole bridges. In turn, the composition and crystal packing of the Mn^II^ salt differs from the rest of the complexes (with six dimethyl ammonia as countercations) in containing one Mn^+2^ and four dimethyl ammonia as countercations. Magnetic data indicate dominant intramolecular antiferromagnetic interactions stabilizing a paramagnetic ground state. Susceptibility data have been successfully modeled with a simple isotropic Hamiltonian for a centrosymmetric linear trimer, H = −2J (S_1_S_2_ + S_2_S_3_) with super-exchange parameters J = −0.4 K for Mn^II^, −7.5 K for Ni^II^ and −45 K for Cu^II^ complex. The magnetic properties of these complexes and their easy processing opens unique possibilities for their incorporation as magnetic molecular probes into such hybrid materials as magnetic/conducting multifunctional materials or as dopant for organic conducting polymers.

## 1. Introduction

Molecule-based magnetism has attracted the attention of a great number of researchers due to its multidisciplinarity and its applications in such fields as (nano)electronics [1,2,3], quantum computing [4], spintronics [5,6] and molecular biology [7,8]. From a more fundamental point of view, molecular magnetism aims at offering a profound comprehension of magneto-structural correlations of molecular magnetic materials. Coordination chemistry appears as a powerful tool to design novel materials with tailor-made magnetic properties [9,10,11,12,13].

In this context, polymetallic complexes are ideal compounds for studying magnetic and electronic interactions between magnetic metal centers in a controlled (molecular) environment. In particular, the investigation of spin coupling effects over long distances has been of high interest for metalloprotein studies [14] and engineering of molecule-based spintronic devices [15]. Exchange spin coupling, or superexchange, is a phenomenon in molecular magnetic systems. The study of magnetic interactions between ions through non-magnetic connecting atoms (linkers) is a challenging task in extended solids [16,17], whereas molecules offer a controlled and tunable framework where spin carriers and linkers can be incorporated as building blocks in the desired connectivity, allowing for analysis based on their localized/molecular nature.

The chemical design and synthesis of bridging ligands, which provides effective pathways to transmit spin coupling effects, promotes an attractive strategy for creating novel and interesting polynuclear complexes with desirable magnetic properties. In particular, 1,2,4-triazole and its derivatives, due to their capacity to form N_1_-N_2_ bridges between metal centers, are interesting ligands for forming stable coordination structures of different dimensionalities, such as discrete polynuclear metal complexes [18,19,20,21], 1D/2D polymers [22,23,24,25,26] or 3D metal–organic frameworks (MOFs) [27,28,29,30,31]. The N_1_-N_2_-1,2,4-triazole bridges offer short and conjugated diatomic pathways to propagate an effective superexchange between the paramagnetic metal centers. Moreover, the nitrogen donor atoms of the triazole ring can create a suitable ligand field for spin transition in ferrous complexes [32,33,34], which provides a potential approach to molecule-based data storage application [35,36,37].

All these reasons justify the use of discrete polynuclear complexes from derivated 1,2,4-triazoles in the study of magnetic interactions between metal centers. In recent years, a large number of polynuclear compounds based on 1,2,4-triazole and its derivatives have been synthetized and investigated to further understand magnetic superexchange coupling [38,39,40].

The chemical nature of the triazole substituents influence the structure and magnetic properties of their complexes [28,29,30,31,32,33,34,35,36,37,38,39,40]. Among the family of 1,2,4-triazoles, the 4-functionalized derivatives have been the most studied for the development of magnetic materials. This type of functionalization does not sterically hinder the N_1_-N_2_ bridging coordination mode, and the functional group might additionally offer different structural motifs to the coordination complexes. In the literature, the most common functional groups in this 4-position are aromatic rings/heterocycles, including pyridyls [41,42,43], triazol [44,45] or tetrazoles [46], and alkyl tails [47]. All of them lead to the formation of neutral ligands, and therefore to the synthesis of cationic coordination metal complexes. Among them, a large variety of triazol-based Fe^II^ systems, including [Fe(4-*R*-1,2,4-triazole)_3_]^2+^ polymers and discrete polynuclear complexes, reveal spin crossover (SCO) behaviour [48,49,50,51].

Recently, our group synthetized a dianionic triazole ligand (L^−2^ = 4-(1,2,4-triazol-4-yl)ethanedisulfonate) with two sulfonated groups in the functional moieties at the 4-position of the triazol. The polar and anionic nature of this ligand would increase solubility and stability in polar solvents of the resulting coordination complexes, facilitating their posterior processing into magnetic hybrid materials. The coordination of this dianionic ligand with iron (II) ions led to the formation of a polyanionic Fe^II^ trinuclear complex with a spin transition above room temperature [52]. Here we present the synthesis, crystal structure and magnetic properties of first-row transition metal complexes (Mn^II^, Co^II^, Ni^II^ and Cu^II^) with this anionic ligand (L), in order to investigate the structure and magnetic behavior of these polyanionic trimers, establishing a rational magneto-structural correlation.

## 2. Results

### 2.1. Synthesis of the Complexes

The complexes were synthesized by reacting the ligand, (Me_2_NH_2_)_6_L (Dimethyl-ammonium 4-(1,2,4-triazol-4-yl)ethanedisulfonate), and the corresponding perchlorate metal salt in water in a 2.5:1 molar ratio, leading to the formation of polyanionic linear trinuclear complexes with the formula [M_3_(μ-L)_6_(H_2_O)_6_]^−6^ [M = Mn^II^ (**Mn**), Co^II^ (**Co**), Ni^II^ (**Ni**), or Cu^II^ (**Cu**)]. These complexes were isolated in solid state as the corresponding dimethyl ammonium salts by slow ethanol vapor diffusion into the aqueous reaction mixture (Figure S1a). This crystallization process yielded needle-shaped crystals for **Mn**, **Co** and **Ni**, with distinct colors depending on the metal: colorless for Mn^II^, orange for Co^II^, and blue-purple for Ni^II^ (Figure S1b). The high quality of these crystals enabled us to determine their crystal structure from single crystal X-Ray diffraction data. We could not isolate Cu complex as single crystals, although highly crystalline powder was obtained, and their crystal lattice was determined by Pawley fit of the X-Ray powder diffractogram.

### 2.2. Structural Characterization

**Figure 1:**
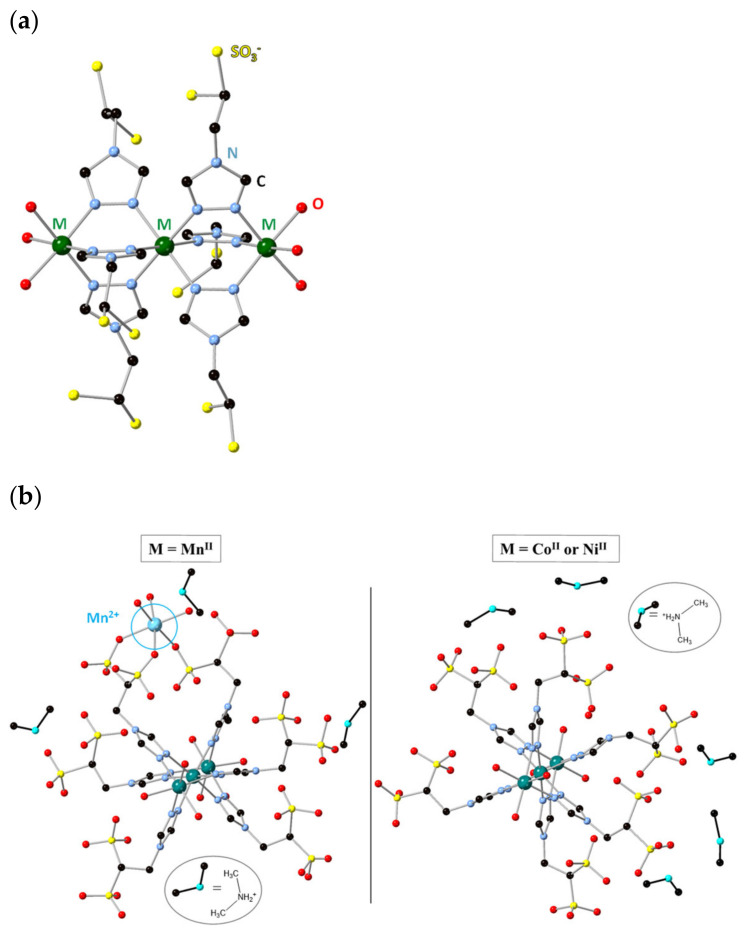
(**a**) Chemical structure of the polyanionic part for all the complexes. Oxygen atoms from the sulfonated groups were omitted for clarity. (**b**) Representation of the anionic part with countercations. Hydrogen atoms are omitted for clarity. Dimethyl ammonium cations (disordered) are represented only in one of their crystallographic positions.

The structure of **Mn**, **Co** and **Ni** was elucidated from single crystal X-Ray diffraction data, collected at 100 K. Crystallographic refinement showed that the three compounds contain the same polyanionic trinuclear units, crystallizing in the triclinic *P*$\bar{1}$ space group (see detailed crystallographic data in Table S1). All of them contain analogous metallic complexes formed by a linear array of three transition metal centers connected by six triazole ligands via two triple N_1_-N_2_ bridges (Figure 1a). These complexes show structure similar to that observed in analogous trinuclear metal complexes based on 4(*R*)-1,2,4-triazole [19]. Thus, the central metal cation is in a MN_6_ octahedral configuration, whereas the terminal metal cations show an MN_3_O_3_ *fac*-octahedral configuration with three nitrogen atoms from the bridging triazole ligands and three oxygen atoms from water molecules occupying the terminal coordination positions. This yields a polyanion with a total charge of −6, given their $\left(M_{3}^{+2}L_{6}^{-2}\right)\cdot$ (H_2_O)_6_ composition. Geometry parameters for these trimers in each compound are summarized in Figure S2 and Table S2. The metal-ligand bonding distances indicate high spin (HS) configurations for all metal positions (average M-N (Å) = 2.239, 2.121 and 2.064 for **Mn**, **Co** and **Ni**, respectively), in good agreement with the corresponding metal ion radii in HS state [*r* (Mn^II^_HS_) = 97 pm, *r* (Co^II^_HS_) = 88 pm and *r* (Ni^II^_HS_) = 83 pm] [52,53].

Although all single crystals confirmed the presence of analogous trimers, the packing is slightly different for **Mn**; whereas **Co** and **Ni** complexes are isostructural with consistent cell parameters and analogous unit cell contents (Table S1), the anionic Mn^II^ trimer crystallizes with a slightly different counter ion content, formed by one Mn^II^ and four [(CH_3_)_2_NH_2_]^+^ moieties. The Mn^2+^ cation external to the trimer occupies a nearby crystallographic position, directly coordinated to three sulfonate groups and completing its coordination geometry with three interstitial water molecules (Figure 1b).

Regarding the crystallographic packing, the trimers are oriented parallel to the *z* axis, forming chains via inter-trimer H-bonds between coordinated water molecules and sulfonated groups (Figure S3). These chains of trimers are also connected to each other along the *x* and *y* axis by additional intermolecular H-bonding interactions [*d*(O···H) = 1.9–2.3 Å for **Mn**; *d*(O···H) = 1.9–2.1 Å for **Co** and **Ni**]. Dimethyl ammonium cations and water molecules are disordered in the interstices of this anionic network.

In order to confirm the phase purity of these compounds in bulk, we analyzed the powder X-ray diffraction (PXRD) data of the polycrystalline samples. Pawley fits were carried out on the experimental diffractograms (Figure S4), obtaining refined cell parameters for each sample (Table S3). The refined cell parameters for **Mn**, **Co** and **Ni** are in good agreement with single crystal data, confirming a single crystallographic phase. The PXRD data for **Cu** was successfully reproduced with a Pawley fitting confirming that this material is isostructural to the Co^II^ and Ni^II^ salts, with consistent cell parameters (Table S3). This is also supported by IR spectroscopy, which shows identical spectra for all the complexes (Figure S5).

### 2.3. Magnetic Measurements

**Figure 2:**
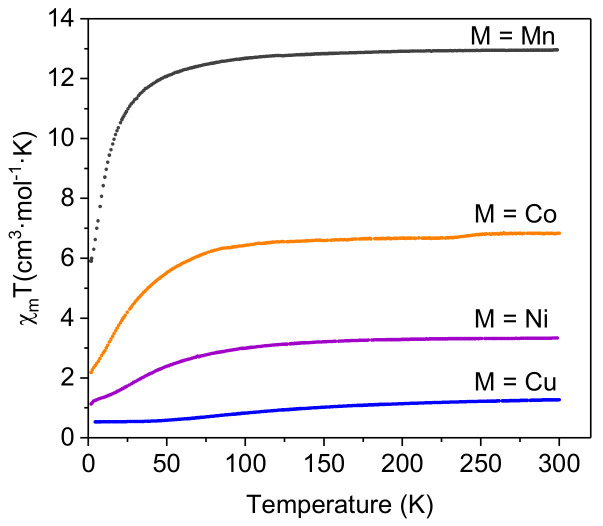
Magnetic susceptibility data for [M_3_(μ-L)_6_(H_2_O)_6_]^−6^ complexes within 300–2 K range.

Magnetic susceptibility (χ_m_) measurements for all the compounds were performed in the 300–2 K range, with an applied magnetic field of 0.1 T (Figure 2). The χ_m_T products of **Mn**, **Co** and **Ni** at room temperature were in good agreement with the expected values for magnetically diluted high spin samples with g ≈ 2 (Table 1). As expected, the Co^II^ trimer exhibited larger χ_m_T values as a result of its high magnetic anisotropy (g > 2), typically found in octahedral Co^II^ ions [54].

Below a certain temperature, the χ_m_T value starts to decrease, suggesting the presence of antiferromagnetic (AF) intramolecular interactions. In good agreement, negative Weiss constants (ϴ) were obtained by fitting the experimental magnetic data (>50 K) to a Curie–Weiss Law (Equations (S1) and (S2), Figure S6 and Table 1).

**Figure 3:**
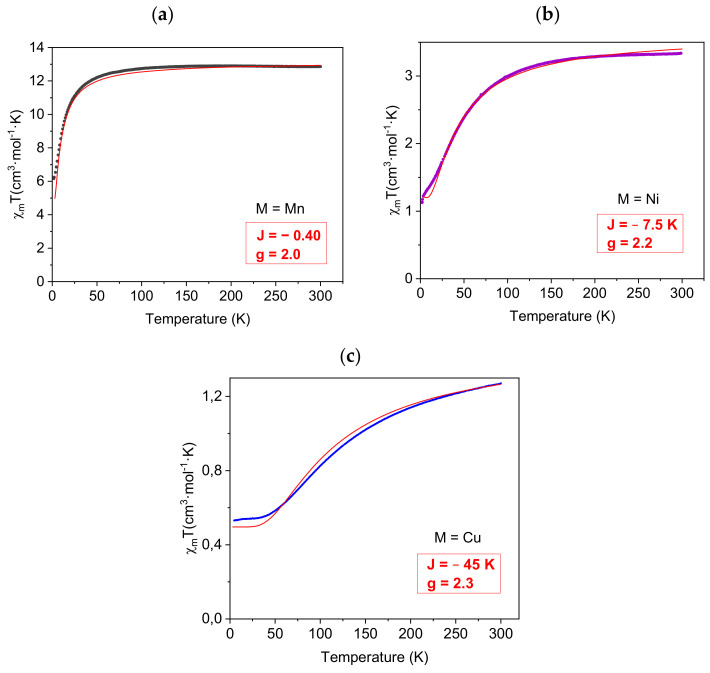
Plots of χ_m_T vs. T for **Mn** (**a**), **Ni** (**b**) and **Cu** (**c**), and their corresponding best-fit curves (red line) obtained with the MAGPACK package.

In order to quantify the intra-trimer spin–spin coupling, the magnetic susceptibility data for **Mn**, **Ni** and **Cu** were modelled, as a first approximation, to a centro-symmetrical linear trimer (Figure S7) by using the isotropic Hamiltonian (Equation (1)):

(1) $$H=ࢤ2J\;s_{2}\;\left(s_{1}+s_{3}\right)$$

where $s_{n}$ are the spins of the different metal centers and J is the corresponding superexchange constant. In a first approximation, intermolecular interactions and zero field splitting contributions are considered to be negligible. The couplings between terminal metal centers are also omitted due to the long metal-to-metal distances (≈7.5 Å) and the inefficient pathway for magnetic exchange between them [55]. Based on this isotropic model, the susceptibility data were successfully reproduced with the MAGPACK package [56,57], obtaining the best fit parameters (J and g) for **Mn**, **Ni** and **Cu** (Figure 3). The magnetic behavior for **Co** could not be modelled with this approximation, and will be discussed later.

In the Mn^II^ compound (Figure 3a), the χ_m_T product at 300 K is 12.94 cm^3^·mol^−1^·K, in agreement with the presence of three high spin Mn^II^ ions with S = 5/2. This value remains constant down to 70 K. At lower temperatures, the χ_m_T product decreases rapidly to reach 5.90 cm^3^·mol^−1^·K at 2 K. This behavior was successfully modelled for the isotropic trinuclear model with best fitting parameters g = 2.0 and J = −0.4 K, which is in good agreement with J values previously reported for triazole-bridged Mn^II^ centers [58,59].

The Ni^II^ compound data (Figure 3b) reveals a χ_m_T value of 3.33 cm^3^·mol^−1^·K at 300 K, which is consistent with the presence of three high spin Ni^II^ ions with S = 1. χ_m_T remains constant down to 150 K, when it starts to decrease quickly down to 1.12 cm^3^·mol^−1^·K at 2 K. The best fitting of these experimental data with the isotropic trimer model yielded g = 2.2 and J = −7.5 K, again in good agreement triazole-bridged Ni^II^ centers (J from −13.8 to −6.7 K) [60,61,62].

**Figure 4:**
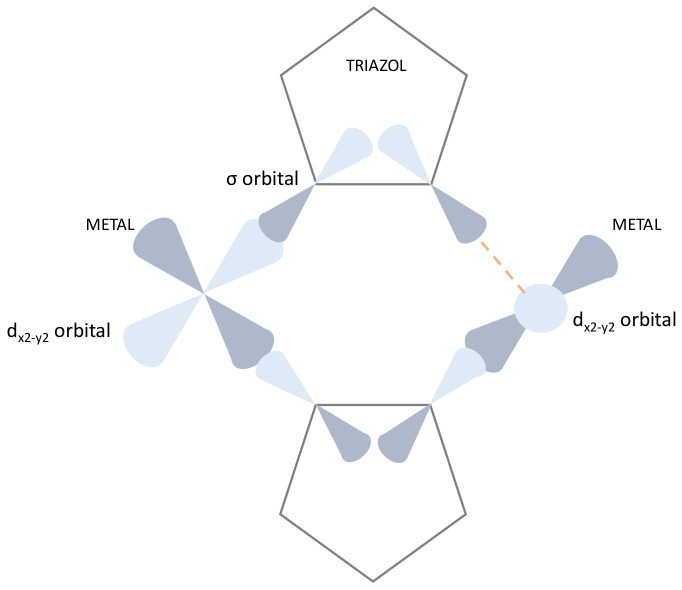
Schematic representation of magnetic molecular orbitals in triazole-bridges complexes. The bridge in the bottom represents the σ overlap for an efficient AF exchange propagation. The bridge in the top represents an absence of orbital overlapping (dotted orange line).

In the Cu^II^ complex (Figure 3c), the χ_m_T product at 300 K is 1.27 cm^3^·mol^−1^·K, which corresponds to the spin-only value for three Cu^II^ ions S = 1/2. Upon being subjected to the cooling process, χ_m_T decreases gradually to reach a constant value around 0.53 cm^3^·mol^−1^·K below 15 K. This experimental data can be well-modelled with J = −45 K and g = 2.3. The value of J parameter is in good agreement with previous reported values for triazole-bridged Cu^II^ centers, where magnetic superexchange only occurs through the N-N bridge that lies in equatorial position with respect to the magnetic dx^2^-y^2^ orbital (Figure 4) [63]. The equatorial configuration of the triazol ensures a good orbital overlap, enhancing the efficient metal-to-metal magnetic interactions [64,65,66]. Consequently, our J value represents weaker antiferromagnetic interaction when compared with the J values between −70 K [67] and −107 K [63] found in analogue Cu^II^ complexes where the bridging modes favor better orbital overlap.

The Co^II^ compound cannot be appropriately modeled with the isotropic model used due to the spin orbit coupling and high single-ion anisotropy [68,69,70]. In the literature, some linear Co^II^ trinuclear complexes with triazole ligands have been modeled with modified Lines approximations [60], showing J value between −6.9 K [55] and −4 K [62].

**Figure 5:**
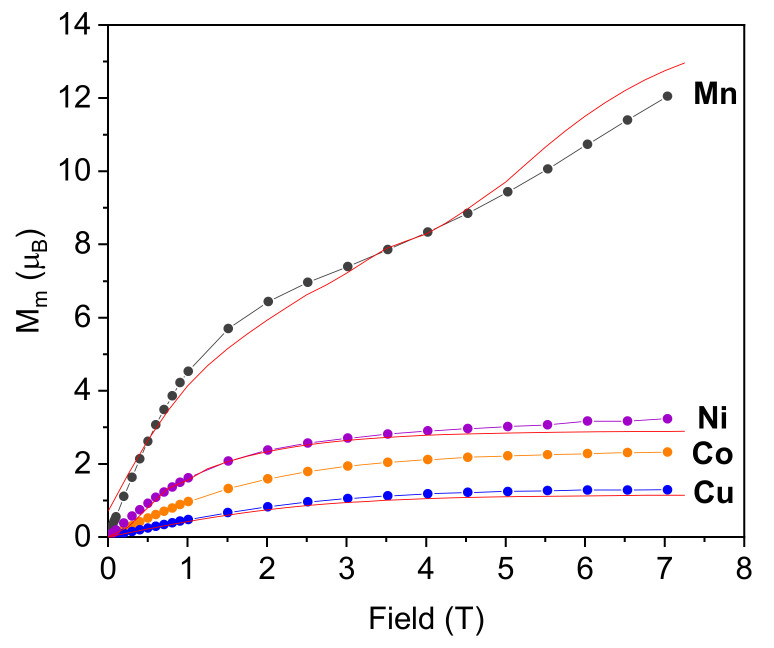
Magnetization (M) vs. magnetic field (H) plots at 2 K for [M_3_(μ-L)_6_(H_2_O)_6_]^−6^ complexes and their corresponding fit curves (red line) obtained with the MAGPACK package, with the resulting parameters of XT vs. T best-fitting.

Field-dependence of the magnetization (M) was also measured at 2 K (Figure 5). The experimental curves are fitted with MAGPACK by using J and g parameters obtained from susceptibility data. The M vs. H curves for all the complexes show a similar field dependence. At low applied fields, the magnetization is proportionally linear to the magnetic field, effectively described by the Curie Law. When the magnetic field becomes larger, the magnetization tends to a saturation value, M_s_ (generally described as Equation (S3)). The saturation values for **Co**, **Ni** and **Cu** are in good agreement with an antiferromagnetic ground state (Table 2). In the case of **Mn**, this initial saturation limit is broken above ≈ 4 T, appearing as a second increase that continues monotonically up to the maximum applied field (7 T) without any sign of reaching a new saturation value.

These results can be rationalized by taking into account the energy levels of the electronic states (Equation (S4), Table S4 and Figure S8). Accordingly, **Mn** exhibits the lowest energy difference between the ground AF state and the excited states. Thus, the applied magnetic field facilitates the population of excited states due to Zeeman splitting, breaking the saturation limit expected for the AF ground state. Even at 7 T, the M_s_ is far from the maximum ferromagnetic alignment. In the rest of the series the magnetic fields applied are not strong enough to provoke the same effect since all samples reach saturation at relatively low applied fields.

## 3. Materials and Methods

### 3.1. Materials and Physical Measurements

All reagents were used as purchased without further purification. Inductively coupled plasma optical emission spectrometry (ICP-OES) analytical data was obtained with an Agilent 755-ES (Santa Clara, CA, USA inductively coupled plasma optical emission spectrometer at the University of Valladolid. Infrared spectroscopy (IR) data were collected with an FTIR Bruker spectrometer (Billerica, MA, USA) model Alpha equipped with an ATR accessory. X-Ray powder diffraction (XRPD) data was collected with a Siemens D5000 diffractometer (Bragg−Brentano parafocusing geometry and vertical θ−θ goniometer, Berlin, Germany) fitted with a curved graphite diffracted-beam monochromator, incident and diffracted-beam Soller slits, a 0.06° receiving slit, and a scintillation counter as a detector. The angular 2θ diffraction range was between 5° and 40°. Data were collected with an angular step of 0.05° at 10 s per step and sample rotation. A low background Si(510) wafer was used as a sample holder. Cu Kα radiation was obtained from a copper X-ray tube operated at 40 kV and 30 mA. The obtained XRPD patterns were analyzed by Pawley profile analysis (between 8° and 35°) using the TOPAS software [71]. A Chebyshev function of seven terms was used to fit the background. Magnetic measurements were carried out on fine-grained powders with a Quantum Design MPMS-XL SQUID magnetometer (Quantum Design, Inc., San Diego, CA, USA). Variable temperature magnetic susceptibility measurements were carried out under an applied field of 1000 Oe at 1 K·min^−1^ between 300 and 2 K. Variable field magnetization measurements were carried out at 2 K from 0 to 7 T.

### 3.2. Synthesis of the Ligand (L)

(Me_2_NH_2_)_2_L (Dimethyl-ammonium 4-(1,2,4-triazol-4-yl)ethanedisulfonate 4-(1,2,4-triazol-4-yl)ethanedisulfonate) was synthesized as previously reported [53].

### 3.3. Synthesis of the Complexes (Mn, Ni and Cu)

(Me_2_NH_2_)_6_[M_3_(μ-L)_6_(H_2_O)_6_]-M = Co^II^ (**Co**), Ni^II^ (**Ni**) and Cu^II^ (**Cu**)- and (Mn)(Me_2_NH_2_)_4_][Mn_3_(μ-L)_6_(H_2_O)_6_] (**Mn**) were synthetized by dissolving M(ClO_4_)_2_·6H_2_O (0.05 mmol) and (Me_2_NH_2_)_6_L (0.05 g; 0.14 mmol) in water and then mixed (total volume 2 mL). Crystals of Mn^II^, Co^II^ and Ni^II^ complexes and crystalline powder of Cu^II^ complex were isolated by slow ethanol vapor diffusion.

**Mn**: IR (ATR): ν = 3420, 3114, 1632, 1205, 1020, 838, 716, 636, 592, 505. ICP experimental and calculated for C_32_H_93_Mn_4_N_22_O_53_S_12_ (2238.72 g/mol) (%_exp_; %_calc_): C (16.99%; 17.16%), H (3.91%; 4.18%), N (14.27%; 13.76%), S (17.31%; 17.18%).

**Co**: IR (ATR): ν = 3420, 3114, 1632, 1205, 1020, 838, 716, 636, 592, 505. Elemental Analysis experimental and calculated for C_36_H_102_Co_3_N_24_O_48_S_12_ (2200.90 g/mol) (%_exp_; %_calc_): C (18.28%; 19.06%), H (4.04%; 4.67%), N (14.10%; 15.27%), S (16.51%; 17.48%).

**Ni**: IR (ATR): ν = 3420, 3121, 1653, 1204, 1020, 844, 715, 639, 595, 505. Elemental Analysis experimental and calculated for C_36_H_100_Ni_3_N_24_O_47_S_12_ (2182.17 g/mol) (%_exp_; %_calc_): C (16.08% 17.06%), H (4.09%; 4.61%), N(15.42%; 15.40%), S (16.14%; 17.63%).

**Cu**: IR (ATR): ν = 3467, 3119, 1643, 1205, 1020, 837, 712, 639, 589, 510. Elemental Analysis experimental and calculated for C_36_H_100_N_24_Cu_3_O_47_S_12_ (2196.73 g/mol) (%_exp_; %_calc_): C (19.30%; 19.68%), H (4.61%; 4.58%), N(15.53%; 15.30%), S (16.61%; 17.51%).

### 3.4. Single Crystal X-ray Diffraction

Data were collected at 100(2) K on a Bruker-Nonius diffractometer (Billerica, MA, USA) with an APPEX 2 4K CCD area detector using Mo–Kα (λ = 0.71073 Å) and equipped with an Oxford Cryostrem 700 plus. Crystal structure solution was obtained using SIR2011 and refinement was performed using SHELXL v. 2018/3 under the ShelXle (Rev. 912) interface. All non-hydrogen atoms were refined anisotropically.

The complexes contain a trinuclear polyanion [M_3_(µ-L)_6_(H_2_O)_6_]^6−^, formed by a linear array of octahedral metal(II) ions (M1-M2-M1) connected by two triple µ-triazole bridges (Figure S2). The central metal positions have a MN_6_ configuration and the terminal metal position completes its N_3_O_3_ hexacoordination with three H_2_O molecules in fac conformation. The asymmetric unit of **Mn** contains one molecule of the Mn^II^ trimer coordinated by six water molecules, four dimethyl ammonium cations, 8.25 non-coordinated water molecules and one manganese cation (+2). This latter is bonded to three water molecules and three sulfonate groups. Most of the sulfonate rests are disordered in different orientations. The dimethylammonium cations are disordered in 11 positions with different occupancies summing a total of four cations. The non-coordinated water molecules are disordered in 14 positions with different occupancies. The asymmetric unit of **Co** and **Ni** contains one molecule of the trinuclear metal complex, six dimethyl ammonium cations and 6.25 and 4.90 water molecules, respectively. Most of the sulfonate rests on the main molecule are disordered in two orientations. The dimethylammonium cations are disordered in 15 positions with different occupancies summing a total of six cations. The non-coordinated water molecules are disordered in 21 and 13 positions with different occupancies. Although the unit cells of these structures are quite similar to **Mn**, they are not isostructural and the gamma angle of the triclinic cell differs from 83.8°/83.6° at **Co**/**Ni** to 87.4° at **Mn**. The structures of **Co** and **Ni** were refined with strong damping factors based on the isostructural iron derivative (CCDC 1016539); after some corrections and omitting the damping factors, refinement remained consistent. The location of the cations and water molecules were extremely diffuse and their positions could only be located using the positions from the iron complex, which offered a much better dataset. Crystallographic data and main refinement parameters are included in Table S1. Crystallographic data for **Mn**, **Co** and **Ni** have been deposited at the Cambridge Crystallographic Database Centre, with deposition numbers CCDC 2045018–2045020, respectively. Copies of this data can be obtained free of charge on application to the CCDC, Cambridge, UK via www.ccdc.cam.ac.uk/data_request/cif.

## 4. Conclusions

A series of complexes based on polyanionic linear trimers, [M_3_(μ-L)_6_(H_2_O)_6_]^−6^ (M = Mn^II^, Co^II^, Ni^II^ and Cu^II^) have been selectively synthesized via the reaction of the ligand L^−2^ = 4-(1,2,4-triazol-4-yl)ethanedisulfonate with the corresponding metal(II) perchlorate salts in a 2.5:1 molar ratio. Crystallographic data confirm identical molecular structure for the polyanionic part of all the complexes, formed by three metal ions linked by triply N-N-triazole bridges. The anionic Co^II^, Ni^II^ and Cu^II^ trimers crystallize with six [(CH_3_)_2_NH_2_]^+^ moieties, whereas the Mn^II^ complex shows a slightly different counter ion content, formed by one Mn^II^ and four [(CH_3_)_2_NH_2_]^+^ moieties. Magnetic susceptibility studies showed dominant intra-trimer antiferromagnetic interactions between terminal and central metal positions, as expected for a N_1_,N_2_-triazole-bridging mode. An isotropic model for centrosymmetric linear trimers was good enough to model the thermal dependence of the magnetic susceptibility, extracting consistent coupling parameters (*J*) with the exception of the Co^II^ derivative, considering intermolecular interactions as negligible. The analysis of the magnetization data at very low temperatures and up to 7 T revealed that the ground antiferromagnetic (AF) state is a good descriptor for these trimers with the exception of the Mn^II^ derivative, where exited states with different multiplicity are close enough in energy to become populated by magnetic fields. Regarding the practical utilization of these complexes, the polyanionic nature of these trimers, along with their high solubility and stability in polar solvents, open interesting possibilities for their incorporation into hybrid materials as magnetic components, such as in magnetic/conducting hybrid salts [72] or as doping elements in organic conducting polymers [73]. These studies are under development.

## Figures and Tables

**Table 1:** Comparison of theoretical and experimental χ_m_T product and Curie–Weiss parameters (*C* and *ϴ*) for all the complexes.

Complex	χ_m_T_theoretical_ (cm^3^·mol^−1^·K)	χ_m_T_experimental_ (cm^3^·mol^−1^·K)	*C* (cm^3^·mol^−1^·K)	*ϴ* (K)
**Mn**	13.12	12.95	13.13	−3.76
**Co**	5.60	6.82	7.07	−11.26
**Ni**	3.00	3.32	3.59	−20.25
**Cu**	1.20	1.26	1.74	−108.48

**Table 2:** Comparison of experimental and theoretical saturation magnetization for the antiferromagnetically (AF) coupled ground state with g = 2 for all the complexes.

Complex	M_s (AF)_ (μ_B_)	M_s (experimental)_ (μ_B_)
Mn	5.00	12.05
Co	4.00	3.22
Ni	3.00	2.32
**Cu**	1.00	1.29

## Data Availability

Not applicable.

## Supplementary Materials

The following are available online. Table S1. Crystallographic data of **Mn**, **Co** and **Ni** crystals; Table S2. Metal-Nitrogen Bond Length for [M_3_(μ-L)_6_(H_2_O)_6_]^−6^ complexes (Figure S2); Table S3. Cell parameters and cell volume obtained from Pawley fit of experimental XRPD pattern shown in Figure S3; Table S4. Energy levels for trimers of **Mn**, **Ni** and **Cu** according to Equation (S4); Figure S1. (a) Pictures of the formation of all the complexes in the synthesis solution after seven days in ethanol vapor flow. (b) Optical microscopic images of **Mn**, **Co** and **Ni** single crystals in their mother liquor; Figure S2. Labeling scheme for the general framework of the [M_3_(μ-L)_6_(H_2_O)_6_]^−6^ complexes. H atoms of coordinating molecules have been omitted for clarity; Figure S3. Packing diagrams showing the arrangement of the trimers. Intratrimer H-bonded interactions (*d*(O···H) = 1.9–2.3/2.1 Å) are represented by red dotted lines for **Mn**/**Co** or **Ni**; Figure S4. Pawley fits of the X-ray powder diffraction patterns of the [M_3_(μ-L)_6_(H_2_O)_6_]^−6^ complexes. The experimental and calculated data are represented as red circles and a black solid line respectively, whereas the green line is the difference between them; Figure S5. Infrarred spectra for MII complexes. The 400–700 cm^−1^ region of the IR spectra is attributed to the metal–ligand stretching vibrations (M-N and M-O vibration modes). The band at 1200 cm^−1^ and 1650 cm^−1^ can be assigned to S=O and C=N stretching, respectively. The bands at 3491 cm^−1^ (OH stretching) and at 1630 cm^−1^ (H-OH bending) evidence the presence of coordinated water molecules; Figure S6. χ^−1^ vs. T plots (solid lines) and their corresponding linear fitting above 50 K (dash lines) for all the complexes; Figure S7. Centrosymmetrical model of linear trinuclear complexes; Figure S8. Energy Diagram for Mn^II^, Ni^II^ and Cu^II^ trimers (see Table S4) with those *J* values determined from experimental χT vs. T data.

## Sample Availability

Samples of the compounds are not available from the authors.
