# Supplementary material for: Crystal Structure and Magnetic Properties of Trinuclear Transition Metal Complexes (MnII, CoII, NiII and CuII) with Bridging Sulfonate-Functionalized 1,2,4-Triazole Derivatives
Source: Molecules. 2021 Oct 4;26(19):6020. doi: 10.3390/molecules26196020 (PMC8512707; doi:10.3390/molecules26196020)

Supporting Information

Crystal structure and magnetic properties of trinuclear transition metal complexes (Mn^{II}, Co^{II}, Ni^{II} and Cu^{II}) with bridging sulfonate-functionalized 1,2,4-triazole derivatives

Andrea Moneo-Corcuera[1*], Breogán Pato-Doldan[1], Irene Sánchez-Molina[1], David Nieto-Castro[1, 2] and José Ramón Galán-Mascarós[1,3*]

Equations

$$\chi = \frac{C}{(T - \theta)} \qquad \text{Equation S1}$$

$$\chi^{-1} = \frac{T}{C} - \frac{\theta}{C} \qquad \text{Equation S2}$$

$$M_{S(\mu_B)} = N \cdot g \cdot S \qquad \text{Equation S3}$$

Where $M_{S(\mu_B)}$ is the saturation magnetization, N is the number of centers with a spin (S) and g-factor (g).

$$E_n = -J(S_T(S_T + 1) - S_A(S_A + 1)) \qquad \text{Equation S4}$$

with $S_A = S_1 + S_3$ and $S_T = S_1 + S_2 + S_3$, and E_n is the energy expressed in function of J.

Table S1. Crystallographic data of **Mn**, **Co** and **Ni** crystals.

	Mn	Co	Ni
Formula	$C_{32}H_{93}Mn_4N_{22}O_{53}S_{12}$	$C_{36}H_{102}Co_3N_{24}O_{48}S_{12}$	$C_{36}H_{100}N_{24}Ni_3O_{47}S_{12}$
Formula weigh	2243.26	2199.12	2180.44
T(K)	100(2)	100(2)	100(2)
Crystal system	Triclinic	Triclinic	Triclinic
Space group	P-1-1	P-1-1	P-1-1
Crystal size	0.05x0.10x0.30	0.20x0.04x0.02	0.20x0.04x0.02
a(Å)	14.3637(2)	14.2894(4)	14.2019(18)
b(Å)	15.5125(2)	15.2780(4)	15.2274(17)
c(Å)	21.5806(3)	21.3682(6)	21.239(2)
α(°)	80.0490(10)	80.9240(10)	81.612(4)
β(°)	85.7230(10)	84.9700(10)	85.261(4)
γ(°)	87.3990(10)	83.7920(10)	83.593(4)
V (Å3)	4720.4(1)	4567.7(2)	4505.2(9)
Z	2	2	2
ρ_{calc} (g·cm^{-3})	1.578	1.599	1.607
μ (mm^{-1})	0.892	0.916	1.002
F (000)	2315	2284	2270
Refl. collected	58741	47861	43963
Ind. reflections	24366	23388	20307
Abs. correction	Multi-scan	Multi-scan	Multi-scan
θ range (°)	1.661-30.344	1.356-29.192	1.687-28.009
Index ranges	$-19 \leq h \leq 19$<br>$-20 \leq k \leq 22$<br>$-28 \leq l \leq 30$	$-14 \leq h \leq 19$<br>$-12 \leq k \leq 20$<br>$-29 \leq l \leq 29$	$-18 \leq h \leq 18$<br>$-20 \leq k \leq 12$<br>$-27 \leq l \leq 27$
Data/restr/para.	24366/ 2030/1748	23388/3474/2033	20307/3444/1986
Goodnesss-of-fit	1.020	1.031	0.996
R1 (I>2σ(I))	0.0789	0.0786	0.0907
wR2 (I>2σ(I))	0.2315	0.2241	0.2340

Table S2. Metal-Nitrogen Bond Length for $[M_3(\mu\text{-}L)_6(H_2O)_6]^{-6}$complexes (Figure S2).

	Mn	Co	Ni
M1-N9	2.266(3)	2.119(4)	2.062(6)
M1-N15	2.254(3)	2.122(4)	2.060(6)
M1-N11	2.240(3)	2.116(4)	2.055(7)
M2-N8	2.240(3)	2.117(4)	2.077(7)
M2-N14	2.222(3)	2.128(4)	2.080(6)
M2-N12	2.210(3)	2.114(4)	2.064(6)
M2-N2	2.265(4)	2.117(4)	2.055(6)
M2-N6	2.232(3)	2.111(4)	2.070(6)
M2-N17	2.226(3)	2.148(4)	2.100(6)
M3-N3	2.275(4)	2.121(4)	2.070(7)
M3-N5	2.275(3)	2.105(4)	2.044(6)
M3-N18	2.231(3)	2.143(4)	2.083(6)

Table S3. Cell parameters and cell volume obtained from Pawley fit of experimental XRPD pattern shown in Figure S3.

Cell Parameters	Mn	Co	Ni	Cu
a (Å)	14.21(2)	14.10(1)	14.19(1)	14.573(5)
b (Å)	15.48(2)	15.44(2)	15.22(2)	14.966(5)
c (Å)	21.62(5)	21.31(2)	21.23(2)	21.216(5)
α (⁰)	80.89(13)	80.99(8)	81.62(5)	81.15(1)
β (⁰)	85.39(18)	84.62(11)	85.25(7)	84.51(2)
γ (⁰)	85.98(10)	83.57(19)	83.57(7)	83.53(3)
V ($Å^3$)	4670(10)	4541(11)	4502(8)	4528(10)

Table S4. Energy levels for trimers of **Mn**, **Ni** and **Cu** according to Equation S4.

Complex	n	S_A	S_T	E_n	
**Mn**	1	5	15/2	–55 J	↑ ↑ ↑
	2	5	13/2	–40 J	
	3	5	11/2	–27 J	
	4	5	9/2	–16 J	
	5	5	7/2	–7 J	
	6	5	5/2	0	↑ ↓ ↑
	7	4	13/2	–50 J	
	8	4	11/2	–37 J	
	9	4	9/2	–26 J	
	10	4	7/2	–17 J	
	11	4	5/2	–10 J	
	12	4	3/2	–5 J	
	13	3	11/2	–45 J	
	14	3	9/2	–34 J	
	15	3	7/2	–25 J	
	16	3	5/2	–18 J	
	17	3	3/2	–13 J	
	18	3	1/2	–10 J	
	19	2	9/2	–40 J	
	20	2	7/2	–31 J	
	21	2	5/2	–24 J	
	22	2	3/2	–19 J	
	23	2	1/2	–16 J	
	24	1	7/2	–35 J	
	25	1	5/2	–28 J	
	26	1	3/2	–23 J	
	27	0	5/2	–30 J	
**Ni**	1	2	3	–10 J	↑ ↑ ↑
	2	2	2	–4 J	
	3	2	1	0	↑ ↓ ↑
	4	1	2	–8 J	
	5	1	1	–4 J	
	6	1	0	–2 J	
	7	0	1	–6 J	
**Cu**	1	1	3/2	–3 J	↑ ↑ ↑
	2	1	1/2	0	↑ ↓ ↑
	3	0	1/2	-2J	

a

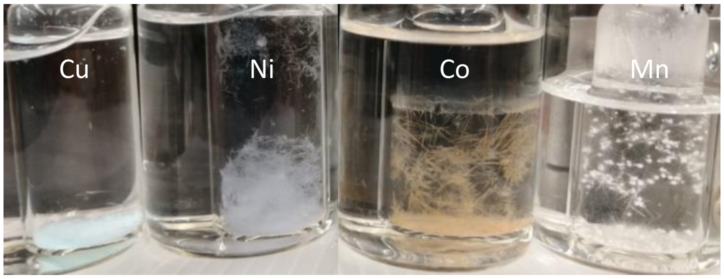

b

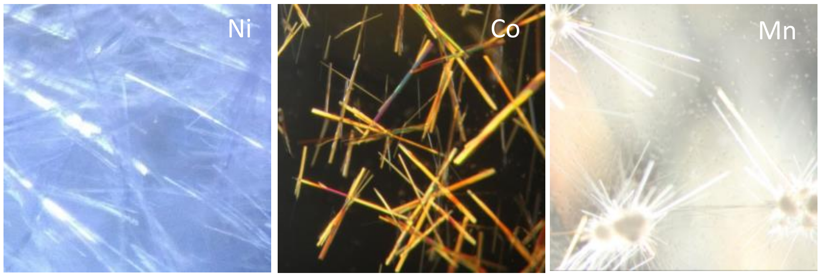

Figure S1. (**a**) Pictures of the formation of all the complexes in the synthesis solution after seven days in ethanol vapor flow. (**b**) Optical microscopic images of **Mn**, **Co** and **Ni** single crystals in their mother liquor.

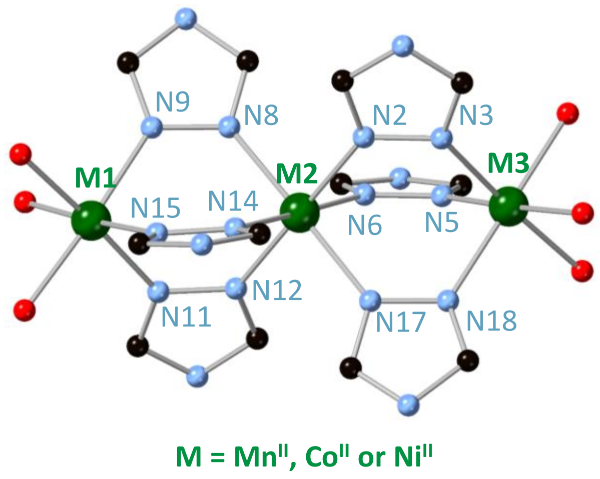

Figure S2. Labeling scheme for the general framework of the $[M_3(\mu\text{-}L)_6(H_2O)_6]^{-6}$complexes. H atoms of coordinating molecules have been omitted for clarity.

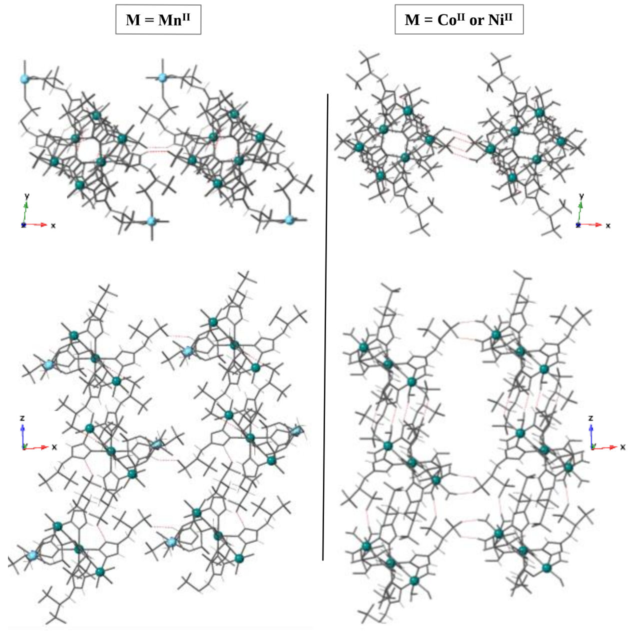

Figure S3. Packing diagrams showing the arrangement of the trimers. Intratrimer H-bonded interactions (d(O···H) = 1.9 – 2.3/2.1 Å) are represented by red dotted lines for **Mn**/**Co** or **Ni**.

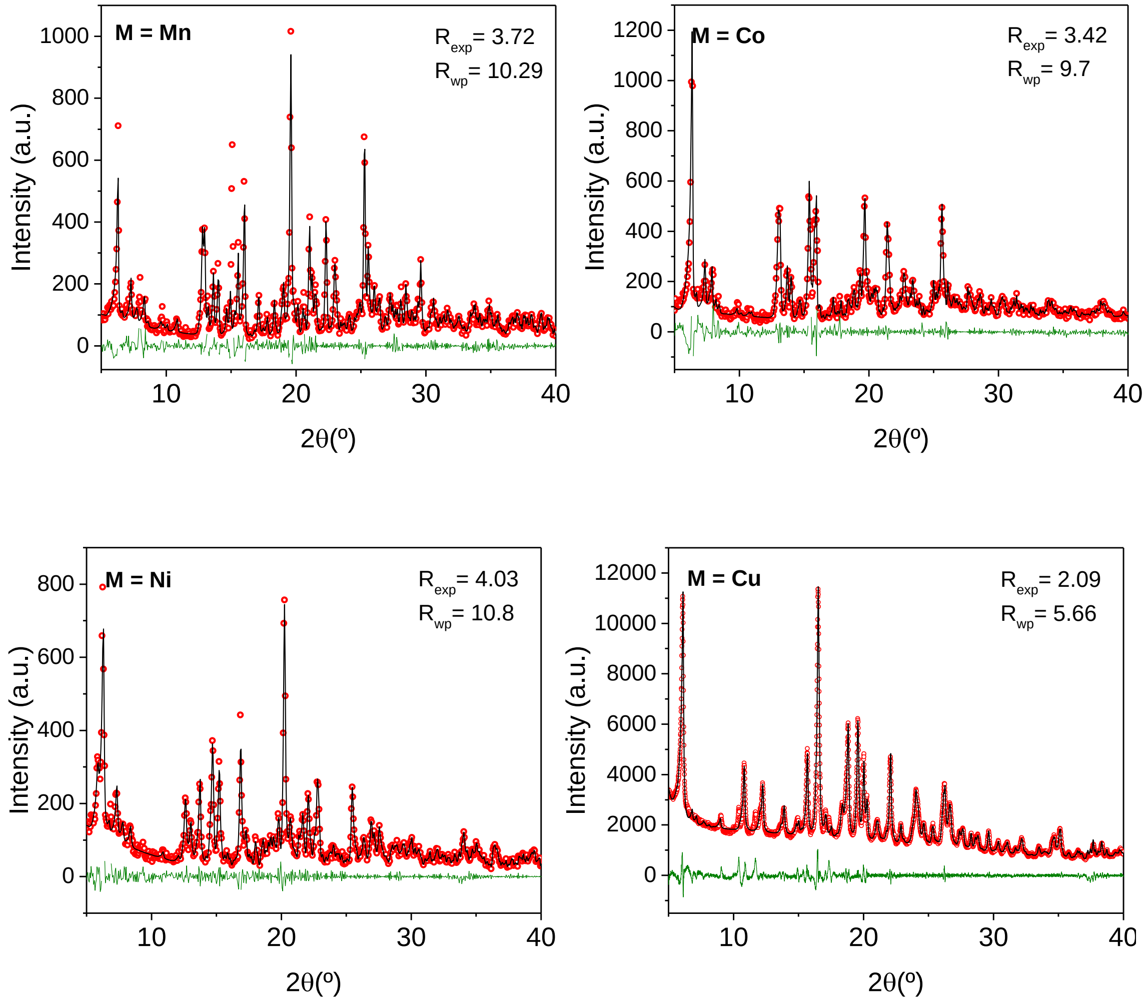

Figure S4. Pawley fits of the X-ray powder diffraction patterns of the $[M_3(\mu\text{-}L)_6(H_2O)_6]^{-6}$ complexes. The experimental and calculated data are represented as red circles and a black solid line respectively, whereas the green line is the difference between them.

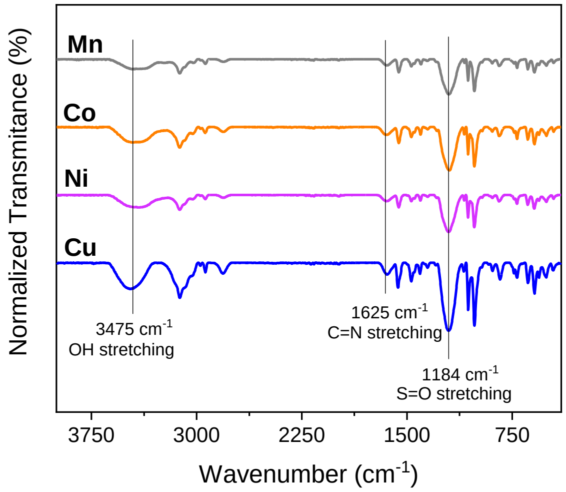

Figure S5. Infrarred spectra for M^{II} complexes. The 400 - 700 cm^{-1} region of the IR spectra is attributed to the metal–ligand stretching vibrations (M-N and M-O vibration modes). The band at 1200 cm^{-1} and 1650 cm^{-1} can be assigned to S=O and C=N stretching, respectively. The bands at 3491 cm^{-1} (OH stretching) and at 1630 cm^{-1} (H-OH bending) evidence the presence of coordinated water molecules.

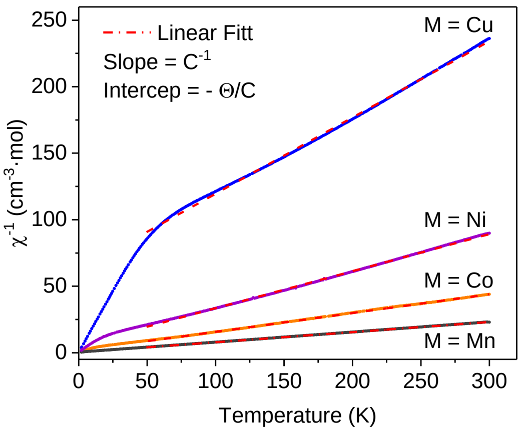

Figure S6. χ^{-1} vs T plots (solid lines) and their corresponding linear fitting above 50 K (dash lines) for all the complexes.

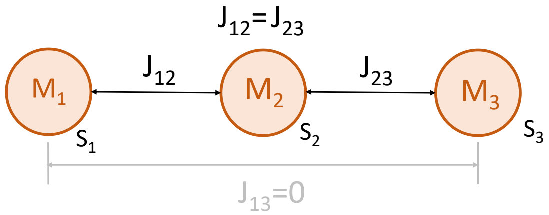

Figure S7. Centrosymmetrical model of linear trinuclear complexes.

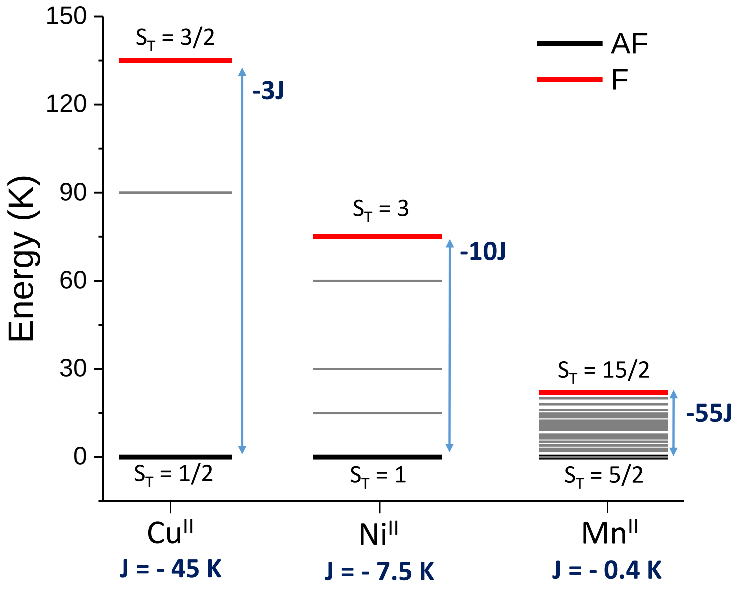

Figure S8. Energy Diagram for Mn^{II}, Ni^{II} and Cu^{II} trimers (see Table S4) with those J values determined from experimental χT *vs* T data.

checkCIF/PLATON report

Structure factors have been supplied for datablock(s) 8-TrMn-96

THIS REPORT IS FOR GUIDANCE ONLY. IF USED AS PART OF A REVIEW PROCEDURE FOR PUBLICATION, IT SHOULD NOT REPLACE THE EXPERTISE OF AN EXPERIENCED CRYSTALLOGRAPHIC REFEREE.

No syntax errors found. CIF dictionary Interpreting this report

Datablock: 8-TrMn-96

Bond precision:	C-C = 0.0064 A		Wavelength=0.71075
Cell:	a=14.3637(2)	b=15.5125(2)	c=21.5806(3)
	alpha=80.049(1)	beta=85.723(1)	gamma=87.399(1)
Temperature:	293 K		

	Calculated	Reported
Volume	4720.41(11)	4720.41(11)
Space group	P -1	P -1
Hall group	-P 1	-P 1
Moiety formula	2(C24 H45 Mn4 N18 O45 S12), 2(C2 H8.50 N O0.25), 0.5(C2 H9 N O)	?
Sum formula	C64 H187 Mn8 N44 O106.50 S24	C32 H93.50 Mn4 N22 O53.25 S12
Mr	4486.54	2243.26
Dx,g cm-3	1.578	1.578
Z	1	2
Mu (mm-1)	0.892	0.892
F000	2315.0	2315.0
F000’	2321.89	
h,k,lmax	20,22,30	19,22,30
Nref	28410	24366
Tmin,Tmax		0.810,1.000
Tmin’		

Correction method= # Reported T Limits: Tmin=0.810 Tmax=1.000
AbsCorr = MULTI-SCAN

Data completeness= 0.858 Theta(max)= 30.344

R(reflections)= 0.0789(18834) wR2(reflections)= 0.2480(24366)

S = 1.020 Npar= 1748

The following ALERTS were generated. Each ALERT has the format
test-name_ALERT_alert-type_alert-level.
Click on the hyperlinks for more details of the test.

Alert level B

PLAT420_ALERT_2_B D-H Without Acceptor O1 --H1O2 . Please Check

Author Response: Due to the high disorder of the water molecules it was not possible to assign correctly all the positions of the hydrogen atoms of these molecules.

PLAT420_ALERT_2_B D-H Without Acceptor O4 --H4O2 . Please Check

Author Response: Due to the high disorder of the water molecules it was not possible to assign correctly all the positions of the hydrogen atoms of these molecules.

PLAT420_ALERT_2_B D-H Without Acceptor O11W --H11L . Please Check

Author Response: Due to the high disorder of the water molecules it was not possible to assign correctly all the positions of the hydrogen atoms of these molecules.

PLAT420_ALERT_2_B D-H Without Acceptor O3W --H3WB . Please Check

Author Response: Due to the high disorder of the water molecules it was not possible to assign correctly all the positions of the hydrogen atoms of these molecules.

PLAT420_ALERT_2_B D-H Without Acceptor O6W --H6WB . Please Check

Author Response: Due to the high disorder of the water molecules it was not possible to assign correctly all the positions of the hydrogen atoms of these molecules.

Alert level C

```
DIFMN02_ALERT_2_C  The minimum difference density is < -0.1*ZMAX*0.75
            _refine_diff_density_min given =     -2.379
            Test value =     -1.875
DIFMN03_ALERT_1_C  The minimum difference density is < -0.1*ZMAX*0.75
            The relevant atom site should be identified.
DIFMX02_ALERT_1_C  The maximum difference density is > 0.1*ZMAX*0.75
            The relevant atom site should be identified.
PLAT029_ALERT_3_C _diffrn_measured_fraction_theta_full value Low .      0.977 Why?
PLAT053_ALERT_1_C Minimum Crystal Dimension Missing (or Error) ...     Please Check
PLAT054_ALERT_1_C Medium  Crystal Dimension Missing (or Error) ...     Please Check
PLAT055_ALERT_1_C Maximum Crystal Dimension Missing (or Error) ...     Please Check
PLAT097_ALERT_2_C Large Reported Max.  (Positive) Residual Density       2.21 eA-3
```

PLAT098_ALERT_2_C Large Reported Min. (Negative) Residual Density   -2.38 eA-3
PLAT220_ALERT_2_C NonSolvent   Resd 1 O   Ueq(max)/Ueq(min) Range   3.5 Ratio
PLAT222_ALERT_3_C NonSolvent Resd 1 H   Uiso(max)/Uiso(min) Range   4.7 Ratio
PLAT223_ALERT_4_C Solv./Anion Resd10 H   Ueq(max)/Ueq(min) Range   4.3 Ratio
PLAT244_ALERT_4_C Low   'Solvent' Ueq as Compared to Neighbors of   N1S Check
PLAT260_ALERT_2_C Large Average Ueq of Residue Including   O7W   0.123 Check
PLAT314_ALERT_2_C Small Angle for H2O: Metal-O3   -H3O1   .   70.12 Degree
PLAT314_ALERT_2_C Small Angle for H2O: Metal-O4   -H4O2   .   85.68 Degree
PLAT314_ALERT_2_C Small Angle for H2O: Metal-O43   -H43V   .   93.03 Degree
PLAT314_ALERT_2_C Small Angle for H2O: Metal-O43   -H43W   .   86.28 Degree
PLAT314_ALERT_2_C Small Angle for H2O: Metal-O44   -H44W   .   80.72 Degree
PLAT314_ALERT_2_C Small Angle for H2O: Metal-O45   -H45V   .   94.34 Degree
PLAT341_ALERT_3_C Low Bond Precision on C-C Bonds ....   0.0064 Ang.
PLAT415_ALERT_2_C Short Inter D-H..H-X   H11A   ..H2WB   .   2.12 Ang.
x,y,z =   1_555 Check
PLAT420_ALERT_2_C D-H Without Acceptor   N1MA   --H1MA   .   Please Check

Author Response: Due to the high disorder of the water molecules it was not possible to assign correctly all the positions of the hydrogen atoms of these molecules.

PLAT906_ALERT_3_C Large K Value in the Analysis of Variance ......   4.218 Check
PLAT911_ALERT_3_C Missing FCF Refl Between Thmin & STh/L=   0.600   385 Report
PLAT918_ALERT_3_C Reflection(s) with I(obs) much Smaller I(calc) .   12 Check
PLAT971_ALERT_2_C Check Calcd Resid. Dens. 0.08A   From C1V   2.19 eA-3
PLAT971_ALERT_2_C Check Calcd Resid. Dens. 0.56A   From C2MA   1.83 eA-3
PLAT971_ALERT_2_C Check Calcd Resid. Dens. 1.01A   From O43   1.77 eA-3
PLAT971_ALERT_2_C Check Calcd Resid. Dens. 1.37A   From C2KA   1.69 eA-3
PLAT971_ALERT_2_C Check Calcd Resid. Dens. 0.76A   From N1V   1.66 eA-3
PLAT971_ALERT_2_C Check Calcd Resid. Dens. 0.51A   From C1MA   1.64 eA-3
PLAT972_ALERT_2_C Check Calcd Resid. Dens. 0.45A   From Mn4   -2.41 eA-3
PLAT972_ALERT_2_C Check Calcd Resid. Dens. 0.40A   From Mn4   -2.36 eA-3
PLAT975_ALERT_2_C Check Calcd Resid. Dens. 0.70A   From O9W   1.49 eA-3
PLAT975_ALERT_2_C Check Calcd Resid. Dens. 0.72A   From O11W   1.38 eA-3
PLAT975_ALERT_2_C Check Calcd Resid. Dens. 0.78A   From O12W   1.38 eA-3
PLAT975_ALERT_2_C Check Calcd Resid. Dens. 0.72A   From O11W   1.28 eA-3
PLAT975_ALERT_2_C Check Calcd Resid. Dens. 0.61A   From O9W   1.19 eA-3
PLAT975_ALERT_2_C Check Calcd Resid. Dens. 0.80A   From O12W   1.14 eA-3
PLAT976_ALERT_2_C Check Calcd Resid. Dens. 0.63A   From O43   -0.86 eA-3
PLAT976_ALERT_2_C Check Calcd Resid. Dens. 0.58A   From O7W   -0.66 eA-3
PLAT976_ALERT_2_C Check Calcd Resid. Dens. 0.54A   From O45   -0.61 eA-3
PLAT976_ALERT_2_C Check Calcd Resid. Dens. 0.49A   From O45   -0.61 eA-3
PLAT976_ALERT_2_C Check Calcd Resid. Dens. 0.55A   From O43   -0.61 eA-3
PLAT976_ALERT_2_C Check Calcd Resid. Dens. 0.47A   From O43   -0.57 eA-3
PLAT976_ALERT_2_C Check Calcd Resid. Dens. 0.46A   From O44   -0.55 eA-3
PLAT976_ALERT_2_C Check Calcd Resid. Dens. 0.50A   From O44   -0.55 eA-3
PLAT977_ALERT_2_C Check Negative Difference Density on H1SB   -0.43 eA-3
PLAT977_ALERT_2_C Check Negative Difference Density on H2SA   -0.35 eA-3
PLAT977_ALERT_2_C Check Negative Difference Density on H2SB   -0.40 eA-3
PLAT977_ALERT_2_C Check Negative Difference Density on H43W   -0.35 eA-3
PLAT977_ALERT_2_C Check Negative Difference Density on H44W   -0.38 eA-3
PLAT977_ALERT_2_C Check Negative Difference Density on H45V   -0.42 eA-3
PLAT977_ALERT_2_C Check Negative Difference Density on H2WB   -0.50 eA-3
PLAT977_ALERT_2_C Check Negative Difference Density on H1OY   -0.31 eA-3
PLAT977_ALERT_2_C Check Negative Difference Density on H2KQ   -0.33 eA-3

Alert level G

PLAT002_ALERT_2_G Number of Distance or Angle Restraints on AtSite   139 Note
PLAT003_ALERT_2_G Number of Uiso or Uij Restrained non-H Atoms ...   110 Report

PLAT007_ALERT_5_G	Number of Unrefined Donor-H Atoms	36	Report
PLAT045_ALERT_1_G	Calculated and Reported Z Differ by a Factor ...	0.50	Check
PLAT083_ALERT_2_G	SHELXL Second Parameter in WGHT Unusually Large	15.64	Why ?
PLAT154_ALERT_1_G	The s.u.'s on the Cell Angles are Equal ..(Note)	0.001	Degree
PLAT171_ALERT_4_G	The CIF-Embedded .res File Contains EADP Records	3	Report
PLAT172_ALERT_4_G	The CIF-Embedded .res File Contains DFIX Records	87	Report
PLAT175_ALERT_4_G	The CIF-Embedded .res File Contains SAME Records	6	Report
PLAT177_ALERT_4_G	The CIF-Embedded .res File Contains DELU Records	16	Report
PLAT178_ALERT_4_G	The CIF-Embedded .res File Contains SIMU Records	16	Report
PLAT186_ALERT_4_G	The CIF-Embedded .res File Contains ISOR Records	22	Report
PLAT199_ALERT_1_G	Reported _cell_measurement_temperature ..... (K)	293	Check
PLAT200_ALERT_1_G	Reported _diffrn_ambient_temperature ..... (K)	293	Check
PLAT230_ALERT_2_G	Hirshfeld Test Diff for S1 --C4 .	7.0	s.u.
PLAT230_ALERT_2_G	Hirshfeld Test Diff for S7 --O25 .	6.5	s.u.
PLAT230_ALERT_2_G	Hirshfeld Test Diff for S1' --C4 .	8.0	s.u.
PLAT232_ALERT_2_G	Hirshfeld Test Diff (M-X) Mn4 --O43 .	8.7	s.u.
PLAT300_ALERT_4_G	Atom Site Occupancy of O13W Constrained at	0.25	Check
PLAT300_ALERT_4_G	Atom Site Occupancy of H13K Constrained at	0.25	Check
PLAT300_ALERT_4_G	Atom Site Occupancy of H13L Constrained at	0.25	Check
PLAT300_ALERT_4_G	Atom Site Occupancy of O12W Constrained at	0.25	Check
PLAT300_ALERT_4_G	Atom Site Occupancy of N1KC Constrained at	0.25	Check
PLAT300_ALERT_4_G	Atom Site Occupancy of C1KC Constrained at	0.25	Check
PLAT300_ALERT_4_G	Atom Site Occupancy of C2KC Constrained at	0.25	Check
PLAT300_ALERT_4_G	Atom Site Occupancy of H12L Constrained at	0.25	Check
PLAT300_ALERT_4_G	Atom Site Occupancy of H1KI Constrained at	0.25	Check
PLAT300_ALERT_4_G	Atom Site Occupancy of H1KQ Constrained at	0.25	Check
PLAT300_ALERT_4_G	Atom Site Occupancy of H1KT Constrained at	0.25	Check
PLAT300_ALERT_4_G	Atom Site Occupancy of H1KR Constrained at	0.25	Check
PLAT300_ALERT_4_G	Atom Site Occupancy of H1KO Constrained at	0.25	Check
PLAT300_ALERT_4_G	Atom Site Occupancy of H2KI Constrained at	0.25	Check
PLAT300_ALERT_4_G	Atom Site Occupancy of H2KQ Constrained at	0.25	Check
PLAT300_ALERT_4_G	Atom Site Occupancy of H2KH Constrained at	0.25	Check
PLAT300_ALERT_4_G	Atom Site Occupancy of N1T Constrained at	0.5	Check
PLAT300_ALERT_4_G	Atom Site Occupancy of C1T Constrained at	0.5	Check
PLAT300_ALERT_4_G	Atom Site Occupancy of C2T Constrained at	0.5	Check
PLAT300_ALERT_4_G	Atom Site Occupancy of H1TA Constrained at	0.5	Check
PLAT300_ALERT_4_G	Atom Site Occupancy of H1TB Constrained at	0.5	Check
PLAT300_ALERT_4_G	Atom Site Occupancy of H1TS Constrained at	0.5	Check
PLAT300_ALERT_4_G	Atom Site Occupancy of H1TT Constrained at	0.5	Check
PLAT300_ALERT_4_G	Atom Site Occupancy of H1TC Constrained at	0.5	Check
PLAT300_ALERT_4_G	Atom Site Occupancy of H2TA Constrained at	0.5	Check
PLAT300_ALERT_4_G	Atom Site Occupancy of H2TB Constrained at	0.5	Check
PLAT300_ALERT_4_G	Atom Site Occupancy of H2TC Constrained at	0.5	Check
PLAT300_ALERT_4_G	Atom Site Occupancy of N1MA Constrained at	0.5	Check
PLAT300_ALERT_4_G	Atom Site Occupancy of C1MA Constrained at	0.5	Check
PLAT300_ALERT_4_G	Atom Site Occupancy of C2MA Constrained at	0.5	Check
PLAT300_ALERT_4_G	Atom Site Occupancy of H1MA Constrained at	0.5	Check
PLAT300_ALERT_4_G	Atom Site Occupancy of H1MB Constrained at	0.5	Check
PLAT300_ALERT_4_G	Atom Site Occupancy of H1MT Constrained at	0.5	Check
PLAT300_ALERT_4_G	Atom Site Occupancy of H1MS Constrained at	0.5	Check
PLAT300_ALERT_4_G	Atom Site Occupancy of H1MR Constrained at	0.5	Check
PLAT300_ALERT_4_G	Atom Site Occupancy of H2MT Constrained at	0.5	Check
PLAT300_ALERT_4_G	Atom Site Occupancy of H2MS Constrained at	0.5	Check
PLAT300_ALERT_4_G	Atom Site Occupancy of H2MR Constrained at	0.5	Check
PLAT300_ALERT_4_G	Atom Site Occupancy of N1OA Constrained at	0.25	Check
PLAT300_ALERT_4_G	Atom Site Occupancy of C1OA Constrained at	0.25	Check
PLAT300_ALERT_4_G	Atom Site Occupancy of C2OA Constrained at	0.25	Check
PLAT300_ALERT_4_G	Atom Site Occupancy of H1OA Constrained at	0.25	Check
PLAT300_ALERT_4_G	Atom Site Occupancy of H1OB Constrained at	0.25	Check
PLAT300_ALERT_4_G	Atom Site Occupancy of H1OC Constrained at	0.25	Check
PLAT300_ALERT_4_G	Atom Site Occupancy of H1OD Constrained at	0.25	Check
PLAT300_ALERT_4_G	Atom Site Occupancy of H1OE Constrained at	0.25	Check

PLAT300_ALERT_4_G Atom Site Occupancy of H2OA Constrained at 0.25 Check
PLAT300_ALERT_4_G Atom Site Occupancy of H2OB Constrained at 0.25 Check
PLAT300_ALERT_4_G Atom Site Occupancy of H2OC Constrained at 0.25 Check
PLAT300_ALERT_4_G Atom Site Occupancy of N1R Constrained at 0.25 Check
PLAT300_ALERT_4_G Atom Site Occupancy of C1R Constrained at 0.25 Check
PLAT300_ALERT_4_G Atom Site Occupancy of C2R Constrained at 0.25 Check
PLAT300_ALERT_4_G Atom Site Occupancy of H1RD Constrained at 0.25 Check
PLAT300_ALERT_4_G Atom Site Occupancy of H1RE Constrained at 0.25 Check
PLAT300_ALERT_4_G Atom Site Occupancy of H1RA Constrained at 0.25 Check
PLAT300_ALERT_4_G Atom Site Occupancy of H1RB Constrained at 0.25 Check
PLAT300_ALERT_4_G Atom Site Occupancy of H1RC Constrained at 0.25 Check
PLAT300_ALERT_4_G Atom Site Occupancy of H2RA Constrained at 0.25 Check
PLAT300_ALERT_4_G Atom Site Occupancy of H2RB Constrained at 0.25 Check
PLAT300_ALERT_4_G Atom Site Occupancy of H2RC Constrained at 0.25 Check
PLAT300_ALERT_4_G Atom Site Occupancy of N1V Constrained at 0.25 Check
PLAT300_ALERT_4_G Atom Site Occupancy of C1V Constrained at 0.25 Check
PLAT300_ALERT_4_G Atom Site Occupancy of C2V Constrained at 0.25 Check
PLAT300_ALERT_4_G Atom Site Occupancy of H1VD Constrained at 0.25 Check
PLAT300_ALERT_4_G Atom Site Occupancy of H1VE Constrained at 0.25 Check
PLAT300_ALERT_4_G Atom Site Occupancy of H1VA Constrained at 0.25 Check
PLAT300_ALERT_4_G Atom Site Occupancy of H1VB Constrained at 0.25 Check
PLAT300_ALERT_4_G Atom Site Occupancy of H1VC Constrained at 0.25 Check
PLAT300_ALERT_4_G Atom Site Occupancy of H2VA Constrained at 0.25 Check
PLAT300_ALERT_4_G Atom Site Occupancy of H2VB Constrained at 0.25 Check
PLAT300_ALERT_4_G Atom Site Occupancy of H2VC Constrained at 0.25 Check
PLAT300_ALERT_4_G Atom Site Occupancy of N1OB Constrained at 0.25 Check
PLAT300_ALERT_4_G Atom Site Occupancy of C1OB Constrained at 0.25 Check
PLAT300_ALERT_4_G Atom Site Occupancy of C2OB Constrained at 0.25 Check
PLAT300_ALERT_4_G Atom Site Occupancy of H1OX Constrained at 0.25 Check
PLAT300_ALERT_4_G Atom Site Occupancy of H1OY Constrained at 0.25 Check
PLAT300_ALERT_4_G Atom Site Occupancy of H1OZ Constrained at 0.25 Check
PLAT300_ALERT_4_G Atom Site Occupancy of H1OF Constrained at 0.25 Check
PLAT300_ALERT_4_G Atom Site Occupancy of H1OG Constrained at 0.25 Check
PLAT300_ALERT_4_G Atom Site Occupancy of H2OX Constrained at 0.25 Check
PLAT300_ALERT_4_G Atom Site Occupancy of H2OY Constrained at 0.25 Check
PLAT300_ALERT_4_G Atom Site Occupancy of H2OZ Constrained at 0.25 Check
PLAT300_ALERT_4_G Atom Site Occupancy of N1MB Constrained at 0.25 Check
PLAT300_ALERT_4_G Atom Site Occupancy of C1MB Constrained at 0.25 Check
PLAT300_ALERT_4_G Atom Site Occupancy of C2MB Constrained at 0.25 Check
PLAT300_ALERT_4_G Atom Site Occupancy of H1MD Constrained at 0.25 Check
PLAT300_ALERT_4_G Atom Site Occupancy of H1ME Constrained at 0.25 Check
PLAT300_ALERT_4_G Atom Site Occupancy of H1MX Constrained at 0.25 Check
PLAT300_ALERT_4_G Atom Site Occupancy of H1MY Constrained at 0.25 Check
PLAT300_ALERT_4_G Atom Site Occupancy of H1MZ Constrained at 0.25 Check
PLAT300_ALERT_4_G Atom Site Occupancy of H2MX Constrained at 0.25 Check
PLAT300_ALERT_4_G Atom Site Occupancy of H2MY Constrained at 0.25 Check
PLAT300_ALERT_4_G Atom Site Occupancy of H2MZ Constrained at 0.25 Check
PLAT300_ALERT_4_G Atom Site Occupancy of N1KA Constrained at 0.25 Check
PLAT300_ALERT_4_G Atom Site Occupancy of C1KA Constrained at 0.25 Check
PLAT300_ALERT_4_G Atom Site Occupancy of C2KA Constrained at 0.25 Check
PLAT300_ALERT_4_G Atom Site Occupancy of H1KD Constrained at 0.25 Check
PLAT300_ALERT_4_G Atom Site Occupancy of H1KE Constrained at 0.25 Check
PLAT300_ALERT_4_G Atom Site Occupancy of H1KX Constrained at 0.25 Check
PLAT300_ALERT_4_G Atom Site Occupancy of H1KY Constrained at 0.25 Check
PLAT300_ALERT_4_G Atom Site Occupancy of H1KZ Constrained at 0.25 Check
PLAT300_ALERT_4_G Atom Site Occupancy of H2KX Constrained at 0.25 Check
PLAT300_ALERT_4_G Atom Site Occupancy of H2KY Constrained at 0.25 Check
PLAT300_ALERT_4_G Atom Site Occupancy of H2KZ Constrained at 0.25 Check
PLAT300_ALERT_4_G Atom Site Occupancy of N1KB Constrained at 0.25 Check
PLAT300_ALERT_4_G Atom Site Occupancy of C1KB Constrained at 0.25 Check
PLAT300_ALERT_4_G Atom Site Occupancy of C2KB Constrained at 0.25 Check
PLAT300_ALERT_4_G Atom Site Occupancy of H1KF Constrained at 0.25 Check

```
PLAT300_ALERT_4_G Atom Site Occupancy of H1KG       Constrained at       0.25 Check
PLAT300_ALERT_4_G Atom Site Occupancy of H1KA       Constrained at       0.25 Check
PLAT300_ALERT_4_G Atom Site Occupancy of H1KB       Constrained at       0.25 Check
PLAT300_ALERT_4_G Atom Site Occupancy of H1KC       Constrained at       0.25 Check
PLAT300_ALERT_4_G Atom Site Occupancy of H2KA       Constrained at       0.25 Check
PLAT300_ALERT_4_G Atom Site Occupancy of H2KB       Constrained at       0.25 Check
PLAT300_ALERT_4_G Atom Site Occupancy of H2KC       Constrained at       0.25 Check
PLAT300_ALERT_4_G Atom Site Occupancy of O3W        Constrained at       0.75 Check
PLAT300_ALERT_4_G Atom Site Occupancy of H3WA       Constrained at       0.75 Check
PLAT300_ALERT_4_G Atom Site Occupancy of H3WB       Constrained at       0.75 Check
PLAT300_ALERT_4_G Atom Site Occupancy of O5W        Constrained at        0.5 Check
PLAT300_ALERT_4_G Atom Site Occupancy of H5WA       Constrained at        0.5 Check
PLAT300_ALERT_4_G Atom Site Occupancy of H5WB       Constrained at        0.5 Check
PLAT300_ALERT_4_G Atom Site Occupancy of O6W        Constrained at        0.5 Check
PLAT300_ALERT_4_G Atom Site Occupancy of H6WA       Constrained at        0.5 Check
PLAT300_ALERT_4_G Atom Site Occupancy of H6WB       Constrained at        0.5 Check
PLAT300_ALERT_4_G Atom Site Occupancy of O8W        Constrained at        0.5 Check
PLAT300_ALERT_4_G Atom Site Occupancy of H8WA       Constrained at        0.5 Check
PLAT300_ALERT_4_G Atom Site Occupancy of H8WB       Constrained at        0.5 Check
PLAT300_ALERT_4_G Atom Site Occupancy of O9W        Constrained at        0.5 Check
PLAT300_ALERT_4_G Atom Site Occupancy of H9WA       Constrained at        0.5 Check
PLAT300_ALERT_4_G Atom Site Occupancy of H9WB       Constrained at        0.5 Check
PLAT300_ALERT_4_G Atom Site Occupancy of O10W       Constrained at        0.5 Check
PLAT300_ALERT_4_G Atom Site Occupancy of H10K       Constrained at        0.5 Check
PLAT300_ALERT_4_G Atom Site Occupancy of H10L       Constrained at        0.5 Check
PLAT300_ALERT_4_G Atom Site Occupancy of O11W       Constrained at        0.5 Check
PLAT300_ALERT_4_G Atom Site Occupancy of H11K       Constrained at        0.5 Check
PLAT300_ALERT_4_G Atom Site Occupancy of H11L       Constrained at        0.5 Check
PLAT300_ALERT_4_G Atom Site Occupancy of H12K       Constrained at       0.25 Check
PLAT301_ALERT_3_G Main Residue  Disorder ..............(Resd  1  )        33% Note
PLAT302_ALERT_4_G Anion/Solvent/Minor-Residue Disorder (Resd  2  )         8% Note
PLAT302_ALERT_4_G Anion/Solvent/Minor-Residue Disorder (Resd  3  )       100% Note
PLAT302_ALERT_4_G Anion/Solvent/Minor-Residue Disorder (Resd  4  )       100% Note
PLAT302_ALERT_4_G Anion/Solvent/Minor-Residue Disorder (Resd  5  )       100% Note
PLAT302_ALERT_4_G Anion/Solvent/Minor-Residue Disorder (Resd  6  )       100% Note
PLAT302_ALERT_4_G Anion/Solvent/Minor-Residue Disorder (Resd  7  )       100% Note
PLAT302_ALERT_4_G Anion/Solvent/Minor-Residue Disorder (Resd  8  )       100% Note
PLAT302_ALERT_4_G Anion/Solvent/Minor-Residue Disorder (Resd  9  )       100% Note
PLAT302_ALERT_4_G Anion/Solvent/Minor-Residue Disorder (Resd 10  )       100% Note
PLAT302_ALERT_4_G Anion/Solvent/Minor-Residue Disorder (Resd 11  )       100% Note
PLAT302_ALERT_4_G Anion/Solvent/Minor-Residue Disorder (Resd 12  )       100% Note
PLAT302_ALERT_4_G Anion/Solvent/Minor-Residue Disorder (Resd 13  )       100% Note
PLAT302_ALERT_4_G Anion/Solvent/Minor-Residue Disorder (Resd 16  )       100% Note
PLAT302_ALERT_4_G Anion/Solvent/Minor-Residue Disorder (Resd 17  )       100% Note
PLAT302_ALERT_4_G Anion/Solvent/Minor-Residue Disorder (Resd 18  )       100% Note
PLAT302_ALERT_4_G Anion/Solvent/Minor-Residue Disorder (Resd 20  )       100% Note
PLAT302_ALERT_4_G Anion/Solvent/Minor-Residue Disorder (Resd 21  )       100% Note
PLAT302_ALERT_4_G Anion/Solvent/Minor-Residue Disorder (Resd 22  )       100% Note
PLAT302_ALERT_4_G Anion/Solvent/Minor-Residue Disorder (Resd 23  )       100% Note
PLAT302_ALERT_4_G Anion/Solvent/Minor-Residue Disorder (Resd 24  )       100% Note
PLAT303_ALERT_2_G Full Occupancy Atom H3O1      with # Connections       2.00 Check
PLAT304_ALERT_4_G Non-Integer Number of Atoms in ..... (Resd  2  )      11.75 Check
PLAT304_ALERT_4_G Non-Integer Number of Atoms in ..... (Resd  3  )       3.25 Check
PLAT304_ALERT_4_G Non-Integer Number of Atoms in ..... (Resd  4  )       5.50 Check
PLAT304_ALERT_4_G Non-Integer Number of Atoms in ..... (Resd  5  )       5.50 Check
PLAT304_ALERT_4_G Non-Integer Number of Atoms in ..... (Resd  6  )       2.75 Check
PLAT304_ALERT_4_G Non-Integer Number of Atoms in ..... (Resd  7  )       2.75 Check
PLAT304_ALERT_4_G Non-Integer Number of Atoms in ..... (Resd  8  )       2.75 Check
PLAT304_ALERT_4_G Non-Integer Number of Atoms in ..... (Resd  9  )       2.75 Check
PLAT304_ALERT_4_G Non-Integer Number of Atoms in ..... (Resd 10  )       2.75 Check
PLAT304_ALERT_4_G Non-Integer Number of Atoms in ..... (Resd 11  )       2.75 Check
PLAT304_ALERT_4_G Non-Integer Number of Atoms in ..... (Resd 12  )       2.75 Check
```

```
PLAT304_ALERT_4_G Non-Integer Number of Atoms in ..... (Resd 13  )       2.34 Check
PLAT304_ALERT_4_G Non-Integer Number of Atoms in ..... (Resd 16  )       2.25 Check
PLAT304_ALERT_4_G Non-Integer Number of Atoms in ..... (Resd 17  )       1.50 Check
PLAT304_ALERT_4_G Non-Integer Number of Atoms in ..... (Resd 18  )       1.50 Check
PLAT304_ALERT_4_G Non-Integer Number of Atoms in ..... (Resd 20  )       1.50 Check
PLAT304_ALERT_4_G Non-Integer Number of Atoms in ..... (Resd 21  )       1.50 Check
PLAT304_ALERT_4_G Non-Integer Number of Atoms in ..... (Resd 22  )       1.50 Check
PLAT304_ALERT_4_G Non-Integer Number of Atoms in ..... (Resd 23  )       1.50 Check
PLAT304_ALERT_4_G Non-Integer Number of Atoms in ..... (Resd 24  )       0.66 Check
PLAT304_ALERT_4_G Non-Integer Number of Atoms in ..... (Resd 25  )       0.25 Check
PLAT367_ALERT_2_G Long?  C(sp?)-C(sp?) Bond  C3       - C4        .       1.54 Ang.
PLAT367_ALERT_2_G Long?  C(sp?)-C(sp?) Bond  C7       - C8        .       1.52 Ang.
PLAT367_ALERT_2_G Long?  C(sp?)-C(sp?) Bond  C16      - C17       .       1.53 Ang.
PLAT413_ALERT_2_G Short Inter XH3 .. XHn     H18      ..H1OX      .       1.74 Ang.
                                                    x,y,z  =     1_555 Check
PLAT413_ALERT_2_G Short Inter XH3 .. XHn     H18      ..H1OY      .       1.86 Ang.
                                                    x,y,z  =     1_555 Check
PLAT413_ALERT_2_G Short Inter XH3 .. XHn     H20A     ..H1OY      .       1.77 Ang.
                                                    x,y,z  =     1_555 Check
PLAT413_ALERT_2_G Short Inter XH3 .. XHn     H20B     ..H1OY      .       2.13 Ang.
                                                    x,y,z  =     1_555 Check
PLAT413_ALERT_2_G Short Inter XH3 .. XHn     H2SA     ..H2KQ      .       2.05 Ang.
                                               -x,2-y,2-z  =     2_577 Check
PLAT413_ALERT_2_G Short Inter XH3 .. XHn     H2SC     ..H1RB      .       2.06 Ang.
                                                  x,1+y,z  =     1_565 Check
PLAT414_ALERT_2_G Short Intra D-H..H-X       H2SA     ..H13L              1.84 Ang.
                                                    x,y,z  =     1_555 Check
PLAT414_ALERT_2_G Short Intra D-H..H-X       H2SB     ..H13K              1.46 Ang.
                                                    x,y,z  =     1_555 Check
PLAT414_ALERT_2_G Short Intra D-H..H-X       H2SB     ..H13L              1.07 Ang.
                                                    x,y,z  =     1_555 Check
PLAT414_ALERT_2_G Short Intra D-H..H-X       H2SC     ..H13K              1.75 Ang.
                                                    x,y,z  =     1_555 Check
PLAT415_ALERT_2_G Short Inter D-H..H-X       H1O1     ..H2MX      .       1.83 Ang.
                                                  x,1+y,z  =     1_565 Check
PLAT415_ALERT_2_G Short Inter D-H..H-X       H1O2     ..H2MX      .       1.73 Ang.
                                                  x,1+y,z  =     1_565 Check
PLAT415_ALERT_2_G Short Inter D-H..H-X       H1O2     ..H2MR      .       1.36 Ang.
                                              1-x,1-y,1-z  =     2_666 Check
PLAT415_ALERT_2_G Short Inter D-H..H-X       H3A      ..H3WA      .       2.14 Ang.
                                                  1+x,y,z  =     1_655 Check
PLAT415_ALERT_2_G Short Inter D-H..H-X       H44V     ..H1KR      .       1.61 Ang.
                                               -x,2-y,2-z  =     2_577 Check
PLAT415_ALERT_2_G Short Inter D-H..H-X       H44W     ..H1KR      .       1.65 Ang.
                                               -x,2-y,2-z  =     2_577 Check
PLAT415_ALERT_2_G Short Inter D-H..H-X       H44W     ..H1KO      .       2.08 Ang.
                                               -x,2-y,2-z  =     2_577 Check
PLAT415_ALERT_2_G Short Inter D-H..H-X       H45V     ..H1VA      .       1.57 Ang.
                                               -x,1-y,1-z  =     2_566 Check
PLAT415_ALERT_2_G Short Inter D-H..H-X       H45W     ..H1VA      .       2.05 Ang.
                                               -x,1-y,1-z  =     2_566 Check
PLAT415_ALERT_2_G Short Inter D-H..H-X       H1WB     ..H1KO      .       2.12 Ang.
                                              1-x,2-y,2-z  =     2_677 Check
PLAT415_ALERT_2_G Short Inter D-H..H-X       H2WA     ..H12K      .       1.67 Ang.
                                              1-x,2-y,2-z  =     2_677 Check
PLAT417_ALERT_2_G Short Inter D-H..H-D       H43V     ..H1KQ      .       1.52 Ang.
                                               -x,2-y,2-z  =     2_577 Check
PLAT417_ALERT_2_G Short Inter D-H..H-D       H44V     ..H5WB      .       2.01 Ang.
                                              -1+x,1+y,z  =     1_465 Check
PLAT417_ALERT_2_G Short Inter D-H..H-D       H45V     ..H1VD      .       1.49 Ang.
                                               -x,1-y,1-z  =     2_566 Check
PLAT417_ALERT_2_G Short Inter D-H..H-D       H45W     ..H9WA      .       1.96 Ang.
```

```
                                   -1+x,1+y,z  =      1_465 Check
PLAT417_ALERT_2_G Short Inter D-H..H-D       H45W     ..H9WB     .       1.85 Ang.
                                   -1+x,1+y,z  =      1_465 Check
PLAT417_ALERT_2_G Short Inter D-H..H-D       H1SB     ..H8WA     .       0.91 Ang.
                                        x,y,z  =      1_555 Check
PLAT417_ALERT_2_G Short Inter D-H..H-D       H1SB     ..H8WB     .       1.83 Ang.
                                        x,y,z  =      1_555 Check
PLAT432_ALERT_2_G Short Inter X...Y Contact  O1       ..C2MB             2.43 Ang.
                                      x,1+y,z  =      1_565 Check
PLAT432_ALERT_2_G Short Inter X...Y Contact  O1       ..C2MA             2.84 Ang.
                                  1-x,1-y,1-z  =      2_666 Check
PLAT432_ALERT_2_G Short Inter X...Y Contact  O1       ..C1MA             3.00 Ang.
                                      x,1+y,z  =      1_565 Check
PLAT432_ALERT_2_G Short Inter X...Y Contact  O5W      ..C2V              2.86 Ang.
                                   1-x,-y,1-z  =      2_656 Check
PLAT432_ALERT_2_G Short Inter X...Y Contact  O6W      ..C2R              2.59 Ang.
                                        x,y,z  =      1_555 Check
PLAT432_ALERT_2_G Short Inter X...Y Contact  O20      ..C1MB             2.86 Ang.
                                      x,1+y,z  =      1_565 Check
PLAT432_ALERT_2_G Short Inter X...Y Contact  O22      ..C2R              2.66 Ang.
                                      x,1+y,z  =      1_565 Check
PLAT432_ALERT_2_G Short Inter X...Y Contact  O26      ..C1               2.99 Ang.
                                  1-x,1-y,2-z  =      2_667 Check
PLAT432_ALERT_2_G Short Inter X...Y Contact  O32      ..C1V              2.69 Ang.
                                      x,1+y,z  =      1_565 Check
PLAT432_ALERT_2_G Short Inter X...Y Contact  O34      ..C2               2.98 Ang.
                                     -1+x,y,z  =      1_455 Check
PLAT432_ALERT_2_G Short Inter X...Y Contact  O44      ..C1KC             2.70 Ang.
                                   -x,2-y,2-z  =      2_577 Check
PLAT432_ALERT_2_G Short Inter X...Y Contact  O45      ..C1V              2.66 Ang.
                                   -x,1-y,1-z  =      2_566 Check
PLAT432_ALERT_2_G Short Inter X...Y Contact  N13      ..C1OB             2.92 Ang.
                                        x,y,z  =      1_555 Check
PLAT432_ALERT_2_G Short Inter X...Y Contact  N14      ..C1OB             2.51 Ang.
                                        x,y,z  =      1_555 Check
PLAT432_ALERT_2_G Short Inter X...Y Contact  N15      ..C1OB             2.03 Ang.
                                        x,y,z  =      1_555 Check
PLAT432_ALERT_2_G Short Inter X...Y Contact  O12W     ..C2KA             2.75 Ang.
                                        x,y,z  =      1_555 Check
PLAT432_ALERT_2_G Short Inter X...Y Contact  O38      ..C5               3.02 Ang.
                                  1-x,1-y,1-z  =      2_666 Check
PLAT432_ALERT_2_G Short Inter X...Y Contact  C1T      ..C1MB             2.75 Ang.
                                        x,y,z  =      1_555 Check
PLAT432_ALERT_2_G Short Inter X...Y Contact  C2T      ..C1OA             2.97 Ang.
                                        x,y,z  =      1_555 Check
PLAT432_ALERT_2_G Short Inter X...Y Contact  C18      ..C1OB             1.90 Ang.
                                        x,y,z  =      1_555 Check
PLAT432_ALERT_2_G Short Inter X...Y Contact  C19      ..C1OB             2.67 Ang.
                                        x,y,z  =      1_555 Check
PLAT432_ALERT_2_G Short Inter X...Y Contact  C20      ..C1OB             2.75 Ang.
                                        x,y,z  =      1_555 Check
PLAT720_ALERT_4_G Number of Unusual/Non-Standard Labels ..........        144 Note
PLAT773_ALERT_2_G Check long C-C Bond in CIF: C18      --C1OB            1.89 Ang.
PLAT789_ALERT_4_G Atoms with Negative _atom_site_disorder_group  #         22 Check
PLAT790_ALERT_4_G Centre of Gravity not Within Unit Cell: Resd.  #         11 Note
              C2 H8 N
PLAT790_ALERT_4_G Centre of Gravity not Within Unit Cell: Resd.  #         20 Note
              H2 O
PLAT790_ALERT_4_G Centre of Gravity not Within Unit Cell: Resd.  #         25 Note
              H
PLAT793_ALERT_4_G Model has Chirality at C12         (Centro SPGR)          R Verify
PLAT794_ALERT_5_G Tentative Bond Valency for Mn2       (II)      .       2.13 Info
```

```
PLAT811_ALERT_5_G No ADDSYM Analysis: Too Many Excluded Atoms ....          ! Info
PLAT860_ALERT_3_G Number of Least-Squares Restraints ............        2030 Note
PLAT883_ALERT_1_G No Info/Value for _atom_sites_solution_primary .     Please Do !
PLAT910_ALERT_3_G Missing # of FCF Reflection(s) Below Theta(Min).          4 Note
PLAT912_ALERT_4_G Missing # of FCF Reflections Above STh/L=  0.600       3573 Note
PLAT933_ALERT_2_G Number of OMIT Records in Embedded .res File ...         17 Note
PLAT941_ALERT_3_G Average HKL Measurement Multiplicity ..........        2.4 Low
PLAT978_ALERT_2_G Number C-C Bonds with Positive Residual Density.          1 Info
PLAT992_ALERT_5_G Repd & Actual _reflns_number_gt Values Differ by          5 Check
```

```
  0 ALERT level A = Most likely a serious problem - resolve or explain
  5 ALERT level B = A potentially serious problem, consider carefully
 57 ALERT level C = Check. Ensure it is not caused by an omission or oversight
268 ALERT level G = General information/check it is not something unexpected

 10 ALERT type 1 CIF construction/syntax error, inconsistent or missing data
113 ALERT type 2 Indicator that the structure model may be wrong or deficient
 10 ALERT type 3 Indicator that the structure quality may be low
193 ALERT type 4 Improvement, methodology, query or suggestion
  4 ALERT type 5 Informative message, check
```

It is advisable to attempt to resolve as many as possible of the alerts in all categories. Often the minor alerts point to easily fixed oversights, errors and omissions in your CIF or refinement strategy, so attention to these fine details can be worthwhile. In order to resolve some of the more serious problems it may be necessary to carry out additional measurements or structure refinements. However, the purpose of your study may justify the reported deviations and the more serious of these should normally be commented upon in the discussion or experimental section of a paper or in the "special_details" fields of the CIF. checkCIF was carefully designed to identify outliers and unusual parameters, but every test has its limitations and alerts that are not important in a particular case may appear. Conversely, the absence of alerts does not guarantee there are no aspects of the results needing attention. It is up to the individual to critically assess their own results and, if necessary, seek expert advice.

Publication of your CIF in IUCr journals

A basic structural check has been run on your CIF. These basic checks will be run on all CIFs submitted for publication in IUCr journals (*Acta Crystallographica*, *Journal of Applied Crystallography*, *Journal of Synchrotron Radiation*); however, if you intend to submit to *Acta Crystallographica Section C* or *E* or *IUCrData*, you should make sure that full publication checks are run on the final version of your CIF prior to submission.

Publication of your CIF in other journals

Please refer to the *Notes for Authors* of the relevant journal for any special instructions relating to CIF submission.

PLATON version of 18/09/2020; check.def file version of 20/08/2020

Datablock 8-TrMn-96 - ellipsoid plot

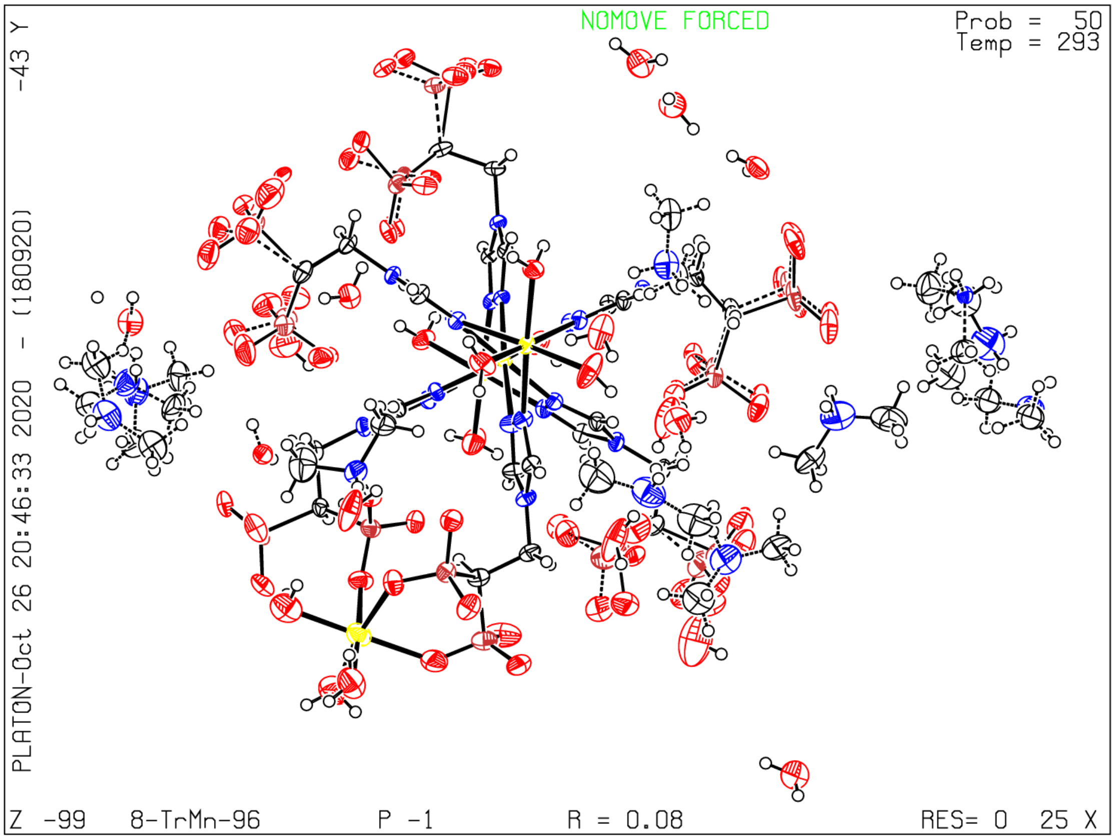

checkCIF/PLATON report

Structure factors have been supplied for datablock(s) mo_TrCo_0m

THIS REPORT IS FOR GUIDANCE ONLY. IF USED AS PART OF A REVIEW PROCEDURE FOR PUBLICATION, IT SHOULD NOT REPLACE THE EXPERTISE OF AN EXPERIENCED CRYSTALLOGRAPHIC REFEREE.

No syntax errors found. CIF dictionary Interpreting this report

Datablock: mo_TrCo_0m

Bond precision: N- C = 0.0064 A Wavelength=0.71073

Cell:	a=14.2894(4)	b=15.2780(4)	c=21.3682(6)
	alpha=80.924(1)	beta=84.970(1)	gamma=83.792(1)
Temperature:	100 K		

	Calculated	Reported
Volume	4567.7(2)	4567.7(2)
Space group	P -1	P -1
Hall group	-P 1	-P 1
Moiety formula	0.20), H O0.50, H O0.50, H O0.50, H O0.50, 2(H0.50 O0.25), H0.5	?
Sum formula	C36 H101.80 Co3 N24 O47.90 S12	C36 H101.80 Co3 N24 O47.90 S12
Mr	2199.13	2199.12
Dx,g cm-3	1.599	1.599
Z	2	2
Mu (mm-1)	0.916	0.916
F000	2284.0	2284.0
F000’	2290.20	
h,k,lmax	19,20,29	19,20,29
Nref	24741	23388
Tmin,Tmax	0.957,0.982	0.554,0.746
Tmin’	0.833	

Correction method= # Reported T Limits: Tmin=0.554 Tmax=0.746
AbsCorr = MULTI-SCAN

Data completeness= 0.945 Theta(max)= 29.192

R(reflections)= 0.0786(14311) wR2(reflections)= 0.2603(23388)

S = 1.031 Npar= 2033

The following ALERTS were generated. Each ALERT has the format
test-name_ALERT_alert-type_alert-level.
Click on the hyperlinks for more details of the test.

Alert level B

PLAT420_ALERT_2_B D-H Without Acceptor O11W --H11V . Please Check

Author Response: Due to the high disorder of the water molecules it was not possible to assign correctly all the positions of the hydrogen atoms of these molecules.

Alert level C

PLAT084_ALERT_3_C High wR2 Value (i.e. > 0.25) .... 0.26 Report
PLAT161_ALERT_4_C Missing or Zero s.u. (esd) on x-coordinate for . C1M Check
PLAT162_ALERT_4_C Missing or Zero s.u. (esd) on y-coordinate for . C1M Check
PLAT163_ALERT_4_C Missing or Zero s.u. (esd) on z-coordinate for . N1H Check
PLAT213_ALERT_2_C Atom O4 has ADP max/min Ratio ..... 3.4 prolat
PLAT220_ALERT_2_C NonSolvent Resd 1 C Ueq(max) / Ueq(min) Range 3.1 Ratio
PLAT220_ALERT_2_C NonSolvent Resd 1 O Ueq(max) / Ueq(min) Range 5.0 Ratio
PLAT220_ALERT_2_C NonSolvent Resd 1 S Ueq(max) / Ueq(min) Range 3.3 Ratio
PLAT223_ALERT_4_C Solv./Anion Resd 5 H Ueq(max)/Ueq(min) Range 4.1 Ratio
PLAT260_ALERT_2_C Large Average Ueq of Residue Including C2C 0.141 Check
PLAT260_ALERT_2_C Large Average Ueq of Residue Including N1A 0.225 Check
PLAT260_ALERT_2_C Large Average Ueq of Residue Including N1E 0.132 Check
PLAT260_ALERT_2_C Large Average Ueq of Residue Including N1H 0.119 Check
PLAT260_ALERT_2_C Large Average Ueq of Residue Including N1F 0.172 Check
PLAT260_ALERT_2_C Large Average Ueq of Residue Including N1M 0.122 Check
PLAT260_ALERT_2_C Large Average Ueq of Residue Including N1O 0.129 Check
PLAT260_ALERT_2_C Large Average Ueq of Residue Including O4W 0.110 Check
PLAT260_ALERT_2_C Large Average Ueq of Residue Including O6W 0.168 Check
PLAT260_ALERT_2_C Large Average Ueq of Residue Including O9W 0.149 Check
PLAT260_ALERT_2_C Large Average Ueq of Residue Including O13W 0.110 Check
PLAT420_ALERT_2_C D-H Without Acceptor N1A --H1A1 . Please Check

Author Response: Due to the high disorder of the water molecules it was not possible to assign correctly all the positions of the hydrogen atoms of these molecules.

PLAT420_ALERT_2_C D-H Without Acceptor N1D --H1D1 . Please Check

Author Response: Due to the high disorder of the water molecules it was not possible to assign correctly all the positions of the hydrogen atoms of these molecules.

PLAT420_ALERT_2_C D-H Without Acceptor N1D --H1D2 . Please Check

Author Response: Due to the high disorder of the water molecules it was not possible to assign correctly all the positions of the hydrogen atoms of these molecules.

```
PLAT420_ALERT_2_C D-H Without Acceptor       N1E      --H1E1     .     Please Check
```

Author Response: Due to the high disorder of the water molecules it was not possible to assign correctly all the positions of the hydrogen atoms of these molecules.

```
PLAT906_ALERT_3_C Large K Value in the Analysis of Variance ......      3.504 Check
PLAT911_ALERT_3_C Missing FCF Refl Between Thmin & STh/L=    0.600        305 Report
PLAT918_ALERT_3_C Reflection(s) with I(obs) much Smaller I(calc) .          3 Check
PLAT977_ALERT_2_C Check Negative Difference Density on H23C             -0.34 eA-3
PLAT977_ALERT_2_C Check Negative Difference Density on H40M             -0.33 eA-3
PLAT977_ALERT_2_C Check Negative Difference Density on H1E1             -0.37 eA-3
PLAT977_ALERT_2_C Check Negative Difference Density on H1E2             -0.36 eA-3
PLAT977_ALERT_2_C Check Negative Difference Density on H1H1             -0.31 eA-3
PLAT977_ALERT_2_C Check Negative Difference Density on H4WA             -0.37 eA-3
PLAT977_ALERT_2_C Check Negative Difference Density on H13V             -0.32 eA-3
PLAT977_ALERT_2_C Check Negative Difference Density on H1M1             -0.32 eA-3
PLAT977_ALERT_2_C Check Negative Difference Density on H5WB             -0.40 eA-3
```

Alert level G

```
PLAT002_ALERT_2_G Number of Distance or Angle Restraints on AtSite        195 Note
PLAT003_ALERT_2_G Number of Uiso or Uij Restrained non-H Atoms ...        178 Report
PLAT004_ALERT_5_G Polymeric Structure Found with Maximum Dimension          1 Info
PLAT007_ALERT_5_G Number of Unrefined Donor-H Atoms ..............         64 Report
PLAT072_ALERT_2_G SHELXL First  Parameter in WGHT  Unusually Large       0.15 Report
PLAT083_ALERT_2_G SHELXL Second Parameter in WGHT  Unusually Large       6.69 Why ?
PLAT154_ALERT_1_G The s.u.'s on the Cell Angles are Equal ..(Note)      0.001 Degree
PLAT172_ALERT_4_G The CIF-Embedded .res File Contains DFIX Records        287 Report
PLAT177_ALERT_4_G The CIF-Embedded .res File Contains DELU Records         10 Report
PLAT178_ALERT_4_G The CIF-Embedded .res File Contains SIMU Records         10 Report
PLAT186_ALERT_4_G The CIF-Embedded .res File Contains ISOR Records         17 Report
PLAT300_ALERT_4_G Atom Site Occupancy of C2C       Constrained at         0.6 Check
PLAT300_ALERT_4_G Atom Site Occupancy of N1N       Constrained at         0.4 Check
PLAT300_ALERT_4_G Atom Site Occupancy of C1N       Constrained at         0.4 Check
PLAT300_ALERT_4_G Atom Site Occupancy of C2N       Constrained at         0.4 Check
PLAT300_ALERT_4_G Atom Site Occupancy of H1N1      Constrained at         0.4 Check
PLAT300_ALERT_4_G Atom Site Occupancy of H1N2      Constrained at         0.4 Check
PLAT300_ALERT_4_G Atom Site Occupancy of H1N3      Constrained at         0.4 Check
PLAT300_ALERT_4_G Atom Site Occupancy of H1N4      Constrained at         0.4 Check
PLAT300_ALERT_4_G Atom Site Occupancy of H1N5      Constrained at         0.4 Check
PLAT300_ALERT_4_G Atom Site Occupancy of H2N1      Constrained at         0.4 Check
PLAT300_ALERT_4_G Atom Site Occupancy of H2N2      Constrained at         0.4 Check
PLAT300_ALERT_4_G Atom Site Occupancy of H2N3      Constrained at         0.4 Check
PLAT300_ALERT_4_G Atom Site Occupancy of N1A       Constrained at         0.5 Check
PLAT300_ALERT_4_G Atom Site Occupancy of C1A       Constrained at         0.5 Check
PLAT300_ALERT_4_G Atom Site Occupancy of C2A       Constrained at         0.5 Check
PLAT300_ALERT_4_G Atom Site Occupancy of H1A1      Constrained at         0.5 Check
PLAT300_ALERT_4_G Atom Site Occupancy of H1A2      Constrained at         0.5 Check
PLAT300_ALERT_4_G Atom Site Occupancy of H1A3      Constrained at         0.5 Check
PLAT300_ALERT_4_G Atom Site Occupancy of H1A4      Constrained at         0.5 Check
PLAT300_ALERT_4_G Atom Site Occupancy of H1A5      Constrained at         0.5 Check
PLAT300_ALERT_4_G Atom Site Occupancy of H2A1      Constrained at         0.5 Check
PLAT300_ALERT_4_G Atom Site Occupancy of H2A2      Constrained at         0.5 Check
PLAT300_ALERT_4_G Atom Site Occupancy of H2A3      Constrained at         0.5 Check
PLAT300_ALERT_4_G Atom Site Occupancy of N1B       Constrained at        0.75 Check
PLAT300_ALERT_4_G Atom Site Occupancy of C1B       Constrained at        0.75 Check
PLAT300_ALERT_4_G Atom Site Occupancy of C2B       Constrained at        0.75 Check
PLAT300_ALERT_4_G Atom Site Occupancy of H1B1      Constrained at        0.75 Check
PLAT300_ALERT_4_G Atom Site Occupancy of H1B2      Constrained at        0.75 Check
```

```
PLAT300_ALERT_4_G Atom Site Occupancy of H1B3       Constrained at       0.75 Check
PLAT300_ALERT_4_G Atom Site Occupancy of H1B4       Constrained at       0.75 Check
PLAT300_ALERT_4_G Atom Site Occupancy of H1B5       Constrained at       0.75 Check
PLAT300_ALERT_4_G Atom Site Occupancy of H2B1       Constrained at       0.75 Check
PLAT300_ALERT_4_G Atom Site Occupancy of H2B2       Constrained at       0.75 Check
PLAT300_ALERT_4_G Atom Site Occupancy of H2B3       Constrained at       0.75 Check
PLAT300_ALERT_4_G Atom Site Occupancy of N1D        Constrained at        0.5 Check
PLAT300_ALERT_4_G Atom Site Occupancy of C1D        Constrained at        0.5 Check
PLAT300_ALERT_4_G Atom Site Occupancy of C2D        Constrained at        0.5 Check
PLAT300_ALERT_4_G Atom Site Occupancy of H1D1       Constrained at        0.5 Check
PLAT300_ALERT_4_G Atom Site Occupancy of H1D2       Constrained at        0.5 Check
PLAT300_ALERT_4_G Atom Site Occupancy of H1D3       Constrained at        0.5 Check
PLAT300_ALERT_4_G Atom Site Occupancy of H1D4       Constrained at        0.5 Check
PLAT300_ALERT_4_G Atom Site Occupancy of H1D5       Constrained at        0.5 Check
PLAT300_ALERT_4_G Atom Site Occupancy of H2D1       Constrained at        0.5 Check
PLAT300_ALERT_4_G Atom Site Occupancy of H2D2       Constrained at        0.5 Check
PLAT300_ALERT_4_G Atom Site Occupancy of H2D3       Constrained at        0.5 Check
PLAT300_ALERT_4_G Atom Site Occupancy of N1E        Constrained at        0.5 Check
PLAT300_ALERT_4_G Atom Site Occupancy of C1E        Constrained at        0.5 Check
PLAT300_ALERT_4_G Atom Site Occupancy of C2E        Constrained at        0.5 Check
PLAT300_ALERT_4_G Atom Site Occupancy of H1E1       Constrained at        0.5 Check
PLAT300_ALERT_4_G Atom Site Occupancy of H1E2       Constrained at        0.5 Check
PLAT300_ALERT_4_G Atom Site Occupancy of H1E3       Constrained at        0.5 Check
PLAT300_ALERT_4_G Atom Site Occupancy of H1E4       Constrained at        0.5 Check
PLAT300_ALERT_4_G Atom Site Occupancy of H1E5       Constrained at        0.5 Check
PLAT300_ALERT_4_G Atom Site Occupancy of H2E1       Constrained at        0.5 Check
PLAT300_ALERT_4_G Atom Site Occupancy of H2E2       Constrained at        0.5 Check
PLAT300_ALERT_4_G Atom Site Occupancy of H2E3       Constrained at        0.5 Check
PLAT300_ALERT_4_G Atom Site Occupancy of N1H        Constrained at        0.5 Check
PLAT300_ALERT_4_G Atom Site Occupancy of C1H        Constrained at        0.5 Check
PLAT300_ALERT_4_G Atom Site Occupancy of C2H        Constrained at        0.5 Check
PLAT300_ALERT_4_G Atom Site Occupancy of H1H1       Constrained at        0.5 Check
PLAT300_ALERT_4_G Atom Site Occupancy of H1H2       Constrained at        0.5 Check
PLAT300_ALERT_4_G Atom Site Occupancy of H1H3       Constrained at        0.5 Check
PLAT300_ALERT_4_G Atom Site Occupancy of H1H4       Constrained at        0.5 Check
PLAT300_ALERT_4_G Atom Site Occupancy of H1H5       Constrained at        0.5 Check
PLAT300_ALERT_4_G Atom Site Occupancy of H2H1       Constrained at        0.5 Check
PLAT300_ALERT_4_G Atom Site Occupancy of H2H2       Constrained at        0.5 Check
PLAT300_ALERT_4_G Atom Site Occupancy of H2H3       Constrained at        0.5 Check
PLAT300_ALERT_4_G Atom Site Occupancy of N1J        Constrained at        0.5 Check
PLAT300_ALERT_4_G Atom Site Occupancy of C1J        Constrained at        0.5 Check
PLAT300_ALERT_4_G Atom Site Occupancy of C2J        Constrained at        0.5 Check
PLAT300_ALERT_4_G Atom Site Occupancy of H1J1       Constrained at        0.5 Check
PLAT300_ALERT_4_G Atom Site Occupancy of H1J2       Constrained at        0.5 Check
PLAT300_ALERT_4_G Atom Site Occupancy of H1J3       Constrained at        0.5 Check
PLAT300_ALERT_4_G Atom Site Occupancy of H1J4       Constrained at        0.5 Check
PLAT300_ALERT_4_G Atom Site Occupancy of H1J5       Constrained at        0.5 Check
PLAT300_ALERT_4_G Atom Site Occupancy of H2J1       Constrained at        0.5 Check
PLAT300_ALERT_4_G Atom Site Occupancy of H2J2       Constrained at        0.5 Check
PLAT300_ALERT_4_G Atom Site Occupancy of H2J3       Constrained at        0.5 Check
PLAT300_ALERT_4_G Atom Site Occupancy of N1F        Constrained at       0.25 Check
PLAT300_ALERT_4_G Atom Site Occupancy of C1F        Constrained at       0.25 Check
PLAT300_ALERT_4_G Atom Site Occupancy of C2F        Constrained at       0.25 Check
PLAT300_ALERT_4_G Atom Site Occupancy of H1F1       Constrained at       0.25 Check
PLAT300_ALERT_4_G Atom Site Occupancy of H1F2       Constrained at       0.25 Check
PLAT300_ALERT_4_G Atom Site Occupancy of H1F3       Constrained at       0.25 Check
PLAT300_ALERT_4_G Atom Site Occupancy of H1F4       Constrained at       0.25 Check
PLAT300_ALERT_4_G Atom Site Occupancy of H1F5       Constrained at       0.25 Check
PLAT300_ALERT_4_G Atom Site Occupancy of H2F1       Constrained at       0.25 Check
PLAT300_ALERT_4_G Atom Site Occupancy of H2F2       Constrained at       0.25 Check
PLAT300_ALERT_4_G Atom Site Occupancy of H2F3       Constrained at       0.25 Check
PLAT300_ALERT_4_G Atom Site Occupancy of N1G        Constrained at       0.25 Check
```

PLAT300_ALERT_4_G Atom Site Occupancy of C1G Constrained at 0.25 Check
PLAT300_ALERT_4_G Atom Site Occupancy of C2G Constrained at 0.25 Check
PLAT300_ALERT_4_G Atom Site Occupancy of H1G1 Constrained at 0.25 Check
PLAT300_ALERT_4_G Atom Site Occupancy of H1G2 Constrained at 0.25 Check
PLAT300_ALERT_4_G Atom Site Occupancy of H1G3 Constrained at 0.25 Check
PLAT300_ALERT_4_G Atom Site Occupancy of H1G4 Constrained at 0.25 Check
PLAT300_ALERT_4_G Atom Site Occupancy of H1G5 Constrained at 0.25 Check
PLAT300_ALERT_4_G Atom Site Occupancy of H2G1 Constrained at 0.25 Check
PLAT300_ALERT_4_G Atom Site Occupancy of H2G2 Constrained at 0.25 Check
PLAT300_ALERT_4_G Atom Site Occupancy of H2G3 Constrained at 0.25 Check
PLAT300_ALERT_4_G Atom Site Occupancy of N1I Constrained at 0.25 Check
PLAT300_ALERT_4_G Atom Site Occupancy of C1I Constrained at 0.25 Check
PLAT300_ALERT_4_G Atom Site Occupancy of C2I Constrained at 0.25 Check
PLAT300_ALERT_4_G Atom Site Occupancy of H1I1 Constrained at 0.25 Check
PLAT300_ALERT_4_G Atom Site Occupancy of H1I2 Constrained at 0.25 Check
PLAT300_ALERT_4_G Atom Site Occupancy of H1I3 Constrained at 0.25 Check
PLAT300_ALERT_4_G Atom Site Occupancy of H1I4 Constrained at 0.25 Check
PLAT300_ALERT_4_G Atom Site Occupancy of H1I5 Constrained at 0.25 Check
PLAT300_ALERT_4_G Atom Site Occupancy of H2I1 Constrained at 0.25 Check
PLAT300_ALERT_4_G Atom Site Occupancy of H2I2 Constrained at 0.25 Check
PLAT300_ALERT_4_G Atom Site Occupancy of H2I3 Constrained at 0.25 Check
PLAT300_ALERT_4_G Atom Site Occupancy of N1K Constrained at 0.25 Check
PLAT300_ALERT_4_G Atom Site Occupancy of C1K Constrained at 0.25 Check
PLAT300_ALERT_4_G Atom Site Occupancy of C2K Constrained at 0.25 Check
PLAT300_ALERT_4_G Atom Site Occupancy of H1K1 Constrained at 0.25 Check
PLAT300_ALERT_4_G Atom Site Occupancy of H1K2 Constrained at 0.25 Check
PLAT300_ALERT_4_G Atom Site Occupancy of H1K3 Constrained at 0.25 Check
PLAT300_ALERT_4_G Atom Site Occupancy of H1K4 Constrained at 0.25 Check
PLAT300_ALERT_4_G Atom Site Occupancy of H1K5 Constrained at 0.25 Check
PLAT300_ALERT_4_G Atom Site Occupancy of H2K1 Constrained at 0.25 Check
PLAT300_ALERT_4_G Atom Site Occupancy of H2K2 Constrained at 0.25 Check
PLAT300_ALERT_4_G Atom Site Occupancy of H2K3 Constrained at 0.25 Check
PLAT300_ALERT_4_G Atom Site Occupancy of N1M Constrained at 0.4 Check
PLAT300_ALERT_4_G Atom Site Occupancy of C1M Constrained at 0.4 Check
PLAT300_ALERT_4_G Atom Site Occupancy of C2M Constrained at 0.4 Check
PLAT300_ALERT_4_G Atom Site Occupancy of H1M1 Constrained at 0.4 Check
PLAT300_ALERT_4_G Atom Site Occupancy of H1M2 Constrained at 0.4 Check
PLAT300_ALERT_4_G Atom Site Occupancy of H1M3 Constrained at 0.4 Check
PLAT300_ALERT_4_G Atom Site Occupancy of H1M4 Constrained at 0.4 Check
PLAT300_ALERT_4_G Atom Site Occupancy of H1M5 Constrained at 0.4 Check
PLAT300_ALERT_4_G Atom Site Occupancy of H2M1 Constrained at 0.4 Check
PLAT300_ALERT_4_G Atom Site Occupancy of H2M2 Constrained at 0.4 Check
PLAT300_ALERT_4_G Atom Site Occupancy of H2M3 Constrained at 0.4 Check
PLAT300_ALERT_4_G Atom Site Occupancy of N1O Constrained at 0.35 Check
PLAT300_ALERT_4_G Atom Site Occupancy of C1O Constrained at 0.35 Check
PLAT300_ALERT_4_G Atom Site Occupancy of C2O Constrained at 0.35 Check
PLAT300_ALERT_4_G Atom Site Occupancy of H1O1 Constrained at 0.35 Check
PLAT300_ALERT_4_G Atom Site Occupancy of H1O2 Constrained at 0.35 Check
PLAT300_ALERT_4_G Atom Site Occupancy of H1O3 Constrained at 0.35 Check
PLAT300_ALERT_4_G Atom Site Occupancy of H1O4 Constrained at 0.35 Check
PLAT300_ALERT_4_G Atom Site Occupancy of H1O5 Constrained at 0.35 Check
PLAT300_ALERT_4_G Atom Site Occupancy of H2O1 Constrained at 0.35 Check
PLAT300_ALERT_4_G Atom Site Occupancy of H2O2 Constrained at 0.35 Check
PLAT300_ALERT_4_G Atom Site Occupancy of H2O3 Constrained at 0.35 Check
PLAT300_ALERT_4_G Atom Site Occupancy of O16W Constrained at 0.2 Check
PLAT300_ALERT_4_G Atom Site Occupancy of H16V Constrained at 0.1 Check
PLAT300_ALERT_4_G Atom Site Occupancy of O4W’ Constrained at 0.5 Check
PLAT300_ALERT_4_G Atom Site Occupancy of H4WC Constrained at 0.5 Check
PLAT300_ALERT_4_G Atom Site Occupancy of H4WD Constrained at 0.5 Check
PLAT300_ALERT_4_G Atom Site Occupancy of O2W Constrained at 0.5 Check
PLAT300_ALERT_4_G Atom Site Occupancy of H2WA Constrained at 0.5 Check
PLAT300_ALERT_4_G Atom Site Occupancy of H2WB Constrained at 0.5 Check

```
PLAT300_ALERT_4_G Atom Site Occupancy of O4W        Constrained at        0.5 Check
PLAT300_ALERT_4_G Atom Site Occupancy of H4WA       Constrained at        0.5 Check
PLAT300_ALERT_4_G Atom Site Occupancy of H4WB       Constrained at        0.5 Check
PLAT300_ALERT_4_G Atom Site Occupancy of O7W        Constrained at        0.5 Check
PLAT300_ALERT_4_G Atom Site Occupancy of H7WA       Constrained at        0.5 Check
PLAT300_ALERT_4_G Atom Site Occupancy of H7WB       Constrained at        0.5 Check
PLAT300_ALERT_4_G Atom Site Occupancy of O5W        Constrained at       0.25 Check
PLAT300_ALERT_4_G Atom Site Occupancy of H5WA       Constrained at       0.25 Check
PLAT300_ALERT_4_G Atom Site Occupancy of H5WB       Constrained at       0.25 Check
PLAT300_ALERT_4_G Atom Site Occupancy of O6W        Constrained at       0.25 Check
PLAT300_ALERT_4_G Atom Site Occupancy of H6WA       Constrained at       0.25 Check
PLAT300_ALERT_4_G Atom Site Occupancy of H6WB       Constrained at       0.25 Check
PLAT300_ALERT_4_G Atom Site Occupancy of O8W        Constrained at       0.25 Check
PLAT300_ALERT_4_G Atom Site Occupancy of H8WA       Constrained at       0.25 Check
PLAT300_ALERT_4_G Atom Site Occupancy of H8WB       Constrained at       0.25 Check
PLAT300_ALERT_4_G Atom Site Occupancy of O9W        Constrained at       0.25 Check
PLAT300_ALERT_4_G Atom Site Occupancy of H9WA       Constrained at       0.25 Check
PLAT300_ALERT_4_G Atom Site Occupancy of H9WB       Constrained at       0.25 Check
PLAT300_ALERT_4_G Atom Site Occupancy of O10W       Constrained at       0.25 Check
PLAT300_ALERT_4_G Atom Site Occupancy of H10U       Constrained at       0.25 Check
PLAT300_ALERT_4_G Atom Site Occupancy of H10V       Constrained at       0.25 Check
PLAT300_ALERT_4_G Atom Site Occupancy of O13W       Constrained at       0.25 Check
PLAT300_ALERT_4_G Atom Site Occupancy of H13U       Constrained at       0.25 Check
PLAT300_ALERT_4_G Atom Site Occupancy of H13V       Constrained at       0.25 Check
PLAT300_ALERT_4_G Atom Site Occupancy of O14W       Constrained at       0.15 Check
PLAT300_ALERT_4_G Atom Site Occupancy of H14U       Constrained at       0.15 Check
PLAT300_ALERT_4_G Atom Site Occupancy of H14V       Constrained at       0.15 Check
PLAT300_ALERT_4_G Atom Site Occupancy of O15W       Constrained at       0.25 Check
PLAT300_ALERT_4_G Atom Site Occupancy of H15U       Constrained at       0.25 Check
PLAT300_ALERT_4_G Atom Site Occupancy of H15V       Constrained at       0.25 Check
PLAT300_ALERT_4_G Atom Site Occupancy of O17W       Constrained at       0.15 Check
PLAT300_ALERT_4_G Atom Site Occupancy of H17U       Constrained at       0.15 Check
PLAT300_ALERT_4_G Atom Site Occupancy of H17V       Constrained at       0.15 Check
PLAT300_ALERT_4_G Atom Site Occupancy of O18W       Constrained at       0.25 Check
PLAT300_ALERT_4_G Atom Site Occupancy of H18U       Constrained at       0.25 Check
PLAT300_ALERT_4_G Atom Site Occupancy of O19W       Constrained at       0.15 Check
PLAT300_ALERT_4_G Atom Site Occupancy of H19U       Constrained at       0.15 Check
PLAT300_ALERT_4_G Atom Site Occupancy of H19V       Constrained at       0.15 Check
PLAT300_ALERT_4_G Atom Site Occupancy of O20W       Constrained at       0.15 Check
PLAT300_ALERT_4_G Atom Site Occupancy of H20U       Constrained at       0.15 Check
PLAT300_ALERT_4_G Atom Site Occupancy of H20V       Constrained at       0.15 Check
PLAT300_ALERT_4_G Atom Site Occupancy of O21W       Constrained at        0.1 Check
PLAT300_ALERT_4_G Atom Site Occupancy of H21U       Constrained at        0.1 Check
PLAT300_ALERT_4_G Atom Site Occupancy of H21V       Constrained at        0.1 Check
PLAT300_ALERT_4_G Atom Site Occupancy of O22W       Constrained at        0.1 Check
PLAT300_ALERT_4_G Atom Site Occupancy of H22U       Constrained at        0.1 Check
PLAT300_ALERT_4_G Atom Site Occupancy of H22V       Constrained at        0.1 Check
PLAT300_ALERT_4_G Atom Site Occupancy of H16U       Constrained at        0.1 Check
PLAT300_ALERT_4_G Atom Site Occupancy of H18V       Constrained at       0.25 Check
PLAT301_ALERT_3_G Main Residue  Disorder .............(Resd  1  )         58% Note
PLAT302_ALERT_4_G Anion/Solvent/Minor-Residue Disorder (Resd  2  )       100% Note
PLAT302_ALERT_4_G Anion/Solvent/Minor-Residue Disorder (Resd  3  )       100% Note
PLAT302_ALERT_4_G Anion/Solvent/Minor-Residue Disorder (Resd  4  )       100% Note
PLAT302_ALERT_4_G Anion/Solvent/Minor-Residue Disorder (Resd  5  )       100% Note
PLAT302_ALERT_4_G Anion/Solvent/Minor-Residue Disorder (Resd  6  )       100% Note
PLAT302_ALERT_4_G Anion/Solvent/Minor-Residue Disorder (Resd  7  )       100% Note
PLAT302_ALERT_4_G Anion/Solvent/Minor-Residue Disorder (Resd  8  )       100% Note
PLAT302_ALERT_4_G Anion/Solvent/Minor-Residue Disorder (Resd  9  )       100% Note
PLAT302_ALERT_4_G Anion/Solvent/Minor-Residue Disorder (Resd 10  )       100% Note
PLAT302_ALERT_4_G Anion/Solvent/Minor-Residue Disorder (Resd 11  )       100% Note
PLAT302_ALERT_4_G Anion/Solvent/Minor-Residue Disorder (Resd 12  )       100% Note
PLAT302_ALERT_4_G Anion/Solvent/Minor-Residue Disorder (Resd 13  )       100% Note
```

```
PLAT302_ALERT_4_G Anion/Solvent/Minor-Residue Disorder (Resd 14  )       100% Note
PLAT302_ALERT_4_G Anion/Solvent/Minor-Residue Disorder (Resd 15  )       100% Note
PLAT302_ALERT_4_G Anion/Solvent/Minor-Residue Disorder (Resd 16  )       100% Note
PLAT302_ALERT_4_G Anion/Solvent/Minor-Residue Disorder (Resd 17  )       100% Note
PLAT302_ALERT_4_G Anion/Solvent/Minor-Residue Disorder (Resd 18  )       100% Note
PLAT302_ALERT_4_G Anion/Solvent/Minor-Residue Disorder (Resd 19  )       100% Note
PLAT302_ALERT_4_G Anion/Solvent/Minor-Residue Disorder (Resd 21  )       100% Note
PLAT302_ALERT_4_G Anion/Solvent/Minor-Residue Disorder (Resd 22  )       100% Note
PLAT302_ALERT_4_G Anion/Solvent/Minor-Residue Disorder (Resd 23  )       100% Note
PLAT302_ALERT_4_G Anion/Solvent/Minor-Residue Disorder (Resd 24  )       100% Note
PLAT302_ALERT_4_G Anion/Solvent/Minor-Residue Disorder (Resd 25  )       100% Note
PLAT302_ALERT_4_G Anion/Solvent/Minor-Residue Disorder (Resd 26  )       100% Note
PLAT302_ALERT_4_G Anion/Solvent/Minor-Residue Disorder (Resd 27  )       100% Note
PLAT302_ALERT_4_G Anion/Solvent/Minor-Residue Disorder (Resd 28  )       100% Note
PLAT302_ALERT_4_G Anion/Solvent/Minor-Residue Disorder (Resd 29  )       100% Note
PLAT302_ALERT_4_G Anion/Solvent/Minor-Residue Disorder (Resd 30  )       100% Note
PLAT302_ALERT_4_G Anion/Solvent/Minor-Residue Disorder (Resd 31  )       100% Note
PLAT302_ALERT_4_G Anion/Solvent/Minor-Residue Disorder (Resd 32  )       100% Note
PLAT302_ALERT_4_G Anion/Solvent/Minor-Residue Disorder (Resd 33  )       100% Note
PLAT302_ALERT_4_G Anion/Solvent/Minor-Residue Disorder (Resd 34  )       100% Note
PLAT415_ALERT_2_G Short Inter D-H..H-X       H11V     ..H1K4     .       1.94 Ang.
                                                   x,y,z  =     1_555 Check
PLAT415_ALERT_2_G Short Inter D-H..H-X       H11V     ..H1K5     .       1.87 Ang.
                                                   x,y,z  =     1_555 Check
PLAT415_ALERT_2_G Short Inter D-H..H-X       H11V     ..H1M4     .       2.06 Ang.
                                              1-x,1-y,-z  =     2_665 Check
PLAT415_ALERT_2_G Short Inter D-H..H-X       H11V     ..H1M5     .       1.63 Ang.
                                              1-x,1-y,-z  =     2_665 Check
PLAT417_ALERT_2_G Short Inter D-H..H-D       H37M     ..H4WA     .       1.97 Ang.
                                                   x,y,z  =     1_555 Check
PLAT417_ALERT_2_G Short Inter D-H..H-D       H37N     ..H4WA     .       0.85 Ang.
                                                   x,y,z  =     1_555 Check
PLAT417_ALERT_2_G Short Inter D-H..H-D       H37N     ..H4WB     .       2.12 Ang.
                                                   x,y,z  =     1_555 Check
PLAT417_ALERT_2_G Short Inter D-H..H-D       H37N     ..H1CB     .       2.12 Ang.
                                                   x,y,z  =     1_555 Check
PLAT417_ALERT_2_G Short Inter D-H..H-D       H42N     ..H2WB     .       1.14 Ang.
                                                 x,1+y,z  =     1_565 Check
PLAT432_ALERT_2_G Short Inter X...Y Contact  O4W'     ..C2C              2.86 Ang.
                                                   x,y,z  =     1_555 Check
PLAT432_ALERT_2_G Short Inter X...Y Contact  O3'      ..C9               3.01 Ang.
                                               -x,2-y,-z  =     2_575 Check
PLAT432_ALERT_2_G Short Inter X...Y Contact  O4W      ..C2C              2.73 Ang.
                                                   x,y,z  =     1_555 Check
PLAT432_ALERT_2_G Short Inter X...Y Contact  O22'     ..C5               2.94 Ang.
                                              -x,2-y,1-z  =     2_576 Check
PLAT432_ALERT_2_G Short Inter X...Y Contact  C19      ..C1K              3.00 Ang.
                                                -1+x,y,z  =     1_455 Check
PLAT432_ALERT_2_G Short Inter X...Y Contact  C19      ..C1M              3.10 Ang.
                                               -x,1-y,-z  =     2_565 Check
PLAT720_ALERT_4_G Number of Unusual/Non-Standard Labels ..........        139 Note
PLAT780_ALERT_1_G Coordinates do not Form a Properly Connected Set     Please Do !
PLAT789_ALERT_4_G Atoms with Negative _atom_site_disorder_group  #         55 Check
PLAT811_ALERT_5_G No ADDSYM Analysis: Too Many Excluded Atoms ....          ! Info
PLAT860_ALERT_3_G Number of Least-Squares Restraints ............        3474 Note
PLAT883_ALERT_1_G No Info/Value for _atom_sites_solution_primary .     Please Do !
PLAT910_ALERT_3_G Missing # of FCF Reflection(s) Below Theta(Min).          1 Note
PLAT912_ALERT_4_G Missing # of FCF Reflections Above STh/L=  0.600       1048 Note
PLAT941_ALERT_3_G Average HKL Measurement Multiplicity ...........        2.0 Low
PLAT965_ALERT_2_G The SHELXL WEIGHT Optimisation has not Converged     Please Check
PLAT992_ALERT_5_G Repd & Actual _reflns_number_gt Values Differ by          3 Check
```

```
  0 ALERT level A = Most likely a serious problem - resolve or explain
  1 ALERT level B = A potentially serious problem, consider carefully
 36 ALERT level C = Check. Ensure it is not caused by an omission or oversight
271 ALERT level G = General information/check it is not something unexpected

  3 ALERT type 1 CIF construction/syntax error, inconsistent or missing data
 49 ALERT type 2 Indicator that the structure model may be wrong or deficient
  8 ALERT type 3 Indicator that the structure quality may be low
244 ALERT type 4 Improvement, methodology, query or suggestion
  4 ALERT type 5 Informative message, check
```

It is advisable to attempt to resolve as many as possible of the alerts in all categories. Often the minor alerts point to easily fixed oversights, errors and omissions in your CIF or refinement strategy, so attention to these fine details can be worthwhile. In order to resolve some of the more serious problems it may be necessary to carry out additional measurements or structure refinements. However, the purpose of your study may justify the reported deviations and the more serious of these should normally be commented upon in the discussion or experimental section of a paper or in the "special_details" fields of the CIF. checkCIF was carefully designed to identify outliers and unusual parameters, but every test has its limitations and alerts that are not important in a particular case may appear. Conversely, the absence of alerts does not guarantee there are no aspects of the results needing attention. It is up to the individual to critically assess their own results and, if necessary, seek expert advice.

Publication of your CIF in IUCr journals

A basic structural check has been run on your CIF. These basic checks will be run on all CIFs submitted for publication in IUCr journals (*Acta Crystallographica*, *Journal of Applied Crystallography*, *Journal of Synchrotron Radiation*); however, if you intend to submit to *Acta Crystallographica Section C* or *E* or *IUCrData*, you should make sure that full publication checks are run on the final version of your CIF prior to submission.

Publication of your CIF in other journals

Please refer to the *Notes for Authors* of the relevant journal for any special instructions relating to CIF submission.

PLATON version of 04/06/2020; check.def file version of 02/06/2020

Datablock mo_TrCo_0m - ellipsoid plot

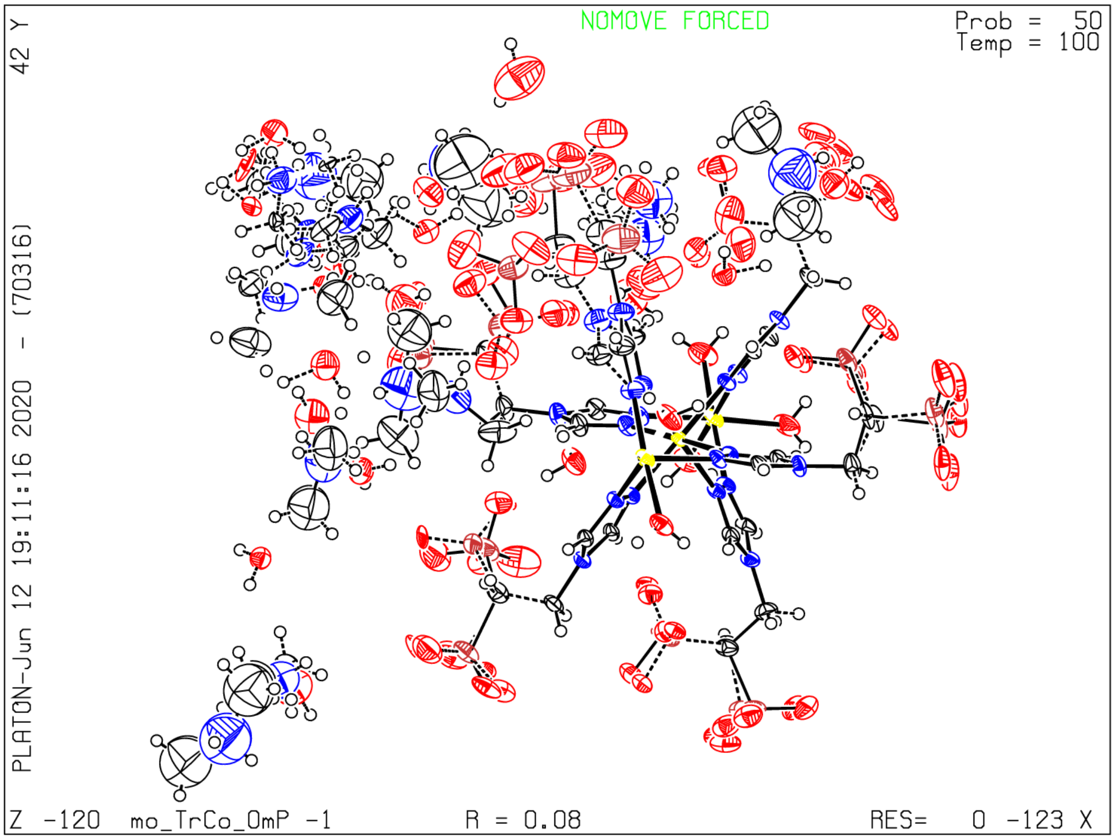

checkCIF/PLATON report

Structure factors have been supplied for datablock(s) mo_TrNi13_0m

THIS REPORT IS FOR GUIDANCE ONLY. IF USED AS PART OF A REVIEW PROCEDURE FOR PUBLICATION, IT SHOULD NOT REPLACE THE EXPERTISE OF AN EXPERIENCED CRYSTALLOGRAPHIC REFEREE.

No syntax errors found. CIF dictionary Interpreting this report

Datablock: mo_TrNi13_0m

```
Bond precision:  N- C = 0.0104 A         Wavelength=0.71073

Cell:           a=14.2019(18)      b=15.2274(17)     c=21.239(2)
                alpha=81.612(4)    beta=85.261(4)    gamma=83.593(4)
Temperature:    100 K
```

	Calculated	Reported
Volume	4505.3(9)	4505.2(9)
Space group	P -1	P -1
Hall group	-P 1	-P 1
Moiety formula	2 N0.25, C0.50 H2 N0.25, C0.50 H2 N0.25, 0.75(C2 H8 N), 1.5(H O	?
Sum formula	C36 H99.80 N24 Ni3 O46.90 S12	C36 H99.80 N24 Ni3 O46.90 S12
Mr	2180.39	2180.44
Dx,g cm-3	1.607	1.607
Z	2	2
Mu (mm-1)	1.002	1.002
F000	2270.0	2270.0
F000’	2276.11	
h,k,lmax	18,20,28	18,20,27
Nref	21758	20307
Tmin,Tmax	0.953,0.980	0.566,0.746
Tmin’	0.818	

```
Correction method= # Reported T Limits: Tmin=0.566 Tmax=0.746
AbsCorr = MULTI-SCAN

Data completeness= 0.933          Theta(max)= 28.009

R(reflections)= 0.0907( 8754)     wR2(reflections)= 0.3065( 20307)

S = 0.996                    Npar= 1986
```

The following ALERTS were generated. Each ALERT has the format
test-name_ALERT_alert-type_alert-level.
Click on the hyperlinks for more details of the test.

Alert level B

```
PLAT420_ALERT_2_B D-H Without Acceptor       O37      --H37N     .     Please Check
```

Author Response: Due to the high disorder level in the structure it was not possible to assign correctly all the contacts, specially the contacts of the water molecules.

Alert level C

```
PLAT026_ALERT_3_C Ratio Observed / Unique Reflections (too) Low ..        43% Check
PLAT029_ALERT_3_C _diffrn_measured_fraction_theta_full value Low .      0.969 Why?
PLAT084_ALERT_3_C High wR2 Value (i.e. > 0.25) ...................       0.31 Report
PLAT161_ALERT_4_C Missing or Zero s.u. (esd) on x-coordinate for .        C1M Check
PLAT162_ALERT_4_C Missing or Zero s.u. (esd) on y-coordinate for .        C1M Check
PLAT220_ALERT_2_C NonSolvent Resd 1  O   Ueq(max) / Ueq(min) Range        4.3 Ratio
PLAT234_ALERT_4_C Large Hirshfeld Difference C7       --C8'      .       0.17 Ang.
PLAT234_ALERT_4_C Large Hirshfeld Difference C7       --C8       .       0.18 Ang.
PLAT260_ALERT_2_C Large Average Ueq of Residue Including       C2C      0.129 Check
PLAT260_ALERT_2_C Large Average Ueq of Residue Including       N1A      0.213 Check
PLAT260_ALERT_2_C Large Average Ueq of Residue Including       N1B      0.121 Check
PLAT260_ALERT_2_C Large Average Ueq of Residue Including       N1D      0.130 Check
PLAT260_ALERT_2_C Large Average Ueq of Residue Including       N1E      0.153 Check
PLAT260_ALERT_2_C Large Average Ueq of Residue Including       N1H      0.106 Check
PLAT260_ALERT_2_C Large Average Ueq of Residue Including       N1J      0.135 Check
PLAT260_ALERT_2_C Large Average Ueq of Residue Including       N1F      0.131 Check
PLAT260_ALERT_2_C Large Average Ueq of Residue Including       N1G      0.143 Check
PLAT260_ALERT_2_C Large Average Ueq of Residue Including       N1I      0.145 Check
PLAT260_ALERT_2_C Large Average Ueq of Residue Including       N1M      0.146 Check
PLAT260_ALERT_2_C Large Average Ueq of Residue Including       N1O      0.215 Check
PLAT260_ALERT_2_C Large Average Ueq of Residue Including      O4W'      0.102 Check
PLAT260_ALERT_2_C Large Average Ueq of Residue Including       O4W      0.105 Check
PLAT260_ALERT_2_C Large Average Ueq of Residue Including       O6W      0.210 Check
PLAT260_ALERT_2_C Large Average Ueq of Residue Including       O9W      0.147 Check
PLAT260_ALERT_2_C Large Average Ueq of Residue Including      O13W      0.132 Check
PLAT260_ALERT_2_C Large Average Ueq of Residue Including      O14W      0.126 Check
PLAT420_ALERT_2_C D-H Without Acceptor       N1A      --H1A1     .     Please Check
```

Author Response: Due to the high disorder level in the structure it was not possible to assign correctly all the contacts, specially the contacts of the water molecules.

```
PLAT420_ALERT_2_C D-H Without Acceptor       N1D      --H1D1     .     Please Check
```

Author Response: Due to the high disorder level in the structure it was not possible to assign correctly all the contacts, specially the contacts of the water molecules.

PLAT420_ALERT_2_C D-H Without Acceptor N1D --H1D2 . Please Check

Author Response: Due to the high disorder level in the structure it was not possible to assign correctly all the contacts, specially the contacts of the water molecules.

PLAT420_ALERT_2_C D-H Without Acceptor N1E --H1E2 . Please Check

Author Response: Due to the high disorder level in the structure it was not possible to assign correctly all the contacts, specially the contacts of the water molecules.

PLAT420_ALERT_2_C D-H Without Acceptor N1J --H1J2 . Please Check

Author Response: Due to the high disorder level in the structure it was not possible to assign correctly all the contacts, specially the contacts of the water molecules.

```
PLAT905_ALERT_3_C Negative K value in the Analysis of Variance ...     -8.375 Report
PLAT911_ALERT_3_C Missing FCF Refl Between Thmin & STh/L=    0.600        503 Report
PLAT973_ALERT_2_C Check Calcd Positive Resid. Density on       Ni3       1.16 eA-3
PLAT975_ALERT_2_C Check Calcd Resid. Dens.  0.51A   From O12W            0.77 eA-3
PLAT975_ALERT_2_C Check Calcd Resid. Dens.  0.41A   From O5W             0.70 eA-3
PLAT977_ALERT_2_C Check Negative Difference Density on H1H1             -0.55 eA-3
PLAT977_ALERT_2_C Check Negative Difference Density on H1H2             -0.40 eA-3
PLAT977_ALERT_2_C Check Negative Difference Density on H2H2             -0.32 eA-3
PLAT977_ALERT_2_C Check Negative Difference Density on H1G1             -0.32 eA-3
PLAT977_ALERT_2_C Check Negative Difference Density on H1K3             -0.36 eA-3
PLAT977_ALERT_2_C Check Negative Difference Density on H1M3             -0.34 eA-3
PLAT977_ALERT_2_C Check Negative Difference Density on H8WA             -0.34 eA-3
```

Alert level G

```
PLAT002_ALERT_2_G Number of Distance or Angle Restraints on AtSite        201 Note
PLAT003_ALERT_2_G Number of Uiso or Uij Restrained non-H Atoms ...        171 Report
PLAT004_ALERT_5_G Polymeric Structure Found with Maximum Dimension          1 Info
PLAT007_ALERT_5_G Number of Unrefined Donor-H Atoms ..............         46 Report
PLAT072_ALERT_2_G SHELXL First  Parameter in WGHT  Unusually Large       0.16 Report
PLAT154_ALERT_1_G The s.u.'s on the Cell Angles are Equal ..(Note)      0.004 Degree
PLAT172_ALERT_4_G The CIF-Embedded .res File Contains DFIX Records        293 Report
PLAT177_ALERT_4_G The CIF-Embedded .res File Contains DELU Records         10 Report
PLAT178_ALERT_4_G The CIF-Embedded .res File Contains SIMU Records         10 Report
PLAT186_ALERT_4_G The CIF-Embedded .res File Contains ISOR Records         14 Report
PLAT300_ALERT_4_G Atom Site Occupancy of C2C        Constrained at        0.6 Check
PLAT300_ALERT_4_G Atom Site Occupancy of N1N        Constrained at        0.4 Check
PLAT300_ALERT_4_G Atom Site Occupancy of C1N        Constrained at        0.4 Check
PLAT300_ALERT_4_G Atom Site Occupancy of C2N        Constrained at        0.4 Check
PLAT300_ALERT_4_G Atom Site Occupancy of H1N1       Constrained at        0.4 Check
PLAT300_ALERT_4_G Atom Site Occupancy of H1N2       Constrained at        0.4 Check
PLAT300_ALERT_4_G Atom Site Occupancy of H1N3       Constrained at        0.4 Check
PLAT300_ALERT_4_G Atom Site Occupancy of H1N4       Constrained at        0.4 Check
PLAT300_ALERT_4_G Atom Site Occupancy of H1N5       Constrained at        0.4 Check
PLAT300_ALERT_4_G Atom Site Occupancy of H2N1       Constrained at        0.4 Check
PLAT300_ALERT_4_G Atom Site Occupancy of H2N2       Constrained at        0.4 Check
PLAT300_ALERT_4_G Atom Site Occupancy of H2N3       Constrained at        0.4 Check
PLAT300_ALERT_4_G Atom Site Occupancy of N1A        Constrained at        0.5 Check
```

PLAT300_ALERT_4_G Atom Site Occupancy of C1A Constrained at 0.5 Check
PLAT300_ALERT_4_G Atom Site Occupancy of C2A Constrained at 0.5 Check
PLAT300_ALERT_4_G Atom Site Occupancy of H1A1 Constrained at 0.5 Check
PLAT300_ALERT_4_G Atom Site Occupancy of H1A2 Constrained at 0.5 Check
PLAT300_ALERT_4_G Atom Site Occupancy of H1A3 Constrained at 0.5 Check
PLAT300_ALERT_4_G Atom Site Occupancy of H1A4 Constrained at 0.5 Check
PLAT300_ALERT_4_G Atom Site Occupancy of H1A5 Constrained at 0.5 Check
PLAT300_ALERT_4_G Atom Site Occupancy of H2A1 Constrained at 0.5 Check
PLAT300_ALERT_4_G Atom Site Occupancy of H2A2 Constrained at 0.5 Check
PLAT300_ALERT_4_G Atom Site Occupancy of H2A3 Constrained at 0.5 Check
PLAT300_ALERT_4_G Atom Site Occupancy of N1B Constrained at 0.75 Check
PLAT300_ALERT_4_G Atom Site Occupancy of C1B Constrained at 0.75 Check
PLAT300_ALERT_4_G Atom Site Occupancy of C2B Constrained at 0.75 Check
PLAT300_ALERT_4_G Atom Site Occupancy of H1B1 Constrained at 0.75 Check
PLAT300_ALERT_4_G Atom Site Occupancy of H1B2 Constrained at 0.75 Check
PLAT300_ALERT_4_G Atom Site Occupancy of H1B3 Constrained at 0.75 Check
PLAT300_ALERT_4_G Atom Site Occupancy of H1B4 Constrained at 0.75 Check
PLAT300_ALERT_4_G Atom Site Occupancy of H1B5 Constrained at 0.75 Check
PLAT300_ALERT_4_G Atom Site Occupancy of H2B1 Constrained at 0.75 Check
PLAT300_ALERT_4_G Atom Site Occupancy of H2B2 Constrained at 0.75 Check
PLAT300_ALERT_4_G Atom Site Occupancy of H2B3 Constrained at 0.75 Check
PLAT300_ALERT_4_G Atom Site Occupancy of N1D Constrained at 0.5 Check
PLAT300_ALERT_4_G Atom Site Occupancy of C1D Constrained at 0.5 Check
PLAT300_ALERT_4_G Atom Site Occupancy of C2D Constrained at 0.5 Check
PLAT300_ALERT_4_G Atom Site Occupancy of H1D1 Constrained at 0.5 Check
PLAT300_ALERT_4_G Atom Site Occupancy of H1D2 Constrained at 0.5 Check
PLAT300_ALERT_4_G Atom Site Occupancy of H1D3 Constrained at 0.5 Check
PLAT300_ALERT_4_G Atom Site Occupancy of H1D4 Constrained at 0.5 Check
PLAT300_ALERT_4_G Atom Site Occupancy of H1D5 Constrained at 0.5 Check
PLAT300_ALERT_4_G Atom Site Occupancy of H2D1 Constrained at 0.5 Check
PLAT300_ALERT_4_G Atom Site Occupancy of H2D2 Constrained at 0.5 Check
PLAT300_ALERT_4_G Atom Site Occupancy of H2D3 Constrained at 0.5 Check
PLAT300_ALERT_4_G Atom Site Occupancy of N1E Constrained at 0.5 Check
PLAT300_ALERT_4_G Atom Site Occupancy of C1E Constrained at 0.5 Check
PLAT300_ALERT_4_G Atom Site Occupancy of C2E Constrained at 0.5 Check
PLAT300_ALERT_4_G Atom Site Occupancy of H1E1 Constrained at 0.5 Check
PLAT300_ALERT_4_G Atom Site Occupancy of H1E2 Constrained at 0.5 Check
PLAT300_ALERT_4_G Atom Site Occupancy of H1E3 Constrained at 0.5 Check
PLAT300_ALERT_4_G Atom Site Occupancy of H1E4 Constrained at 0.5 Check
PLAT300_ALERT_4_G Atom Site Occupancy of H1E5 Constrained at 0.5 Check
PLAT300_ALERT_4_G Atom Site Occupancy of H2E1 Constrained at 0.5 Check
PLAT300_ALERT_4_G Atom Site Occupancy of H2E2 Constrained at 0.5 Check
PLAT300_ALERT_4_G Atom Site Occupancy of H2E3 Constrained at 0.5 Check
PLAT300_ALERT_4_G Atom Site Occupancy of N1H Constrained at 0.5 Check
PLAT300_ALERT_4_G Atom Site Occupancy of C1H Constrained at 0.5 Check
PLAT300_ALERT_4_G Atom Site Occupancy of C2H Constrained at 0.5 Check
PLAT300_ALERT_4_G Atom Site Occupancy of H1H1 Constrained at 0.5 Check
PLAT300_ALERT_4_G Atom Site Occupancy of H1H2 Constrained at 0.5 Check
PLAT300_ALERT_4_G Atom Site Occupancy of H1H3 Constrained at 0.5 Check
PLAT300_ALERT_4_G Atom Site Occupancy of H1H4 Constrained at 0.5 Check
PLAT300_ALERT_4_G Atom Site Occupancy of H1H5 Constrained at 0.5 Check
PLAT300_ALERT_4_G Atom Site Occupancy of H2H1 Constrained at 0.5 Check
PLAT300_ALERT_4_G Atom Site Occupancy of H2H2 Constrained at 0.5 Check
PLAT300_ALERT_4_G Atom Site Occupancy of H2H3 Constrained at 0.5 Check
PLAT300_ALERT_4_G Atom Site Occupancy of N1J Constrained at 0.5 Check
PLAT300_ALERT_4_G Atom Site Occupancy of C1J Constrained at 0.5 Check
PLAT300_ALERT_4_G Atom Site Occupancy of C2J Constrained at 0.5 Check
PLAT300_ALERT_4_G Atom Site Occupancy of H1J1 Constrained at 0.5 Check
PLAT300_ALERT_4_G Atom Site Occupancy of H1J2 Constrained at 0.5 Check
PLAT300_ALERT_4_G Atom Site Occupancy of H1J3 Constrained at 0.5 Check
PLAT300_ALERT_4_G Atom Site Occupancy of H1J4 Constrained at 0.5 Check
PLAT300_ALERT_4_G Atom Site Occupancy of H1J5 Constrained at 0.5 Check

PLAT300_ALERT_4_G Atom Site Occupancy of H2J1 Constrained at 0.5 Check
PLAT300_ALERT_4_G Atom Site Occupancy of H2J2 Constrained at 0.5 Check
PLAT300_ALERT_4_G Atom Site Occupancy of H2J3 Constrained at 0.5 Check
PLAT300_ALERT_4_G Atom Site Occupancy of N1F Constrained at 0.25 Check
PLAT300_ALERT_4_G Atom Site Occupancy of C1F Constrained at 0.25 Check
PLAT300_ALERT_4_G Atom Site Occupancy of C2F Constrained at 0.25 Check
PLAT300_ALERT_4_G Atom Site Occupancy of H1F1 Constrained at 0.25 Check
PLAT300_ALERT_4_G Atom Site Occupancy of H1F2 Constrained at 0.25 Check
PLAT300_ALERT_4_G Atom Site Occupancy of H1F3 Constrained at 0.25 Check
PLAT300_ALERT_4_G Atom Site Occupancy of H1F4 Constrained at 0.25 Check
PLAT300_ALERT_4_G Atom Site Occupancy of H1F5 Constrained at 0.25 Check
PLAT300_ALERT_4_G Atom Site Occupancy of H2F1 Constrained at 0.25 Check
PLAT300_ALERT_4_G Atom Site Occupancy of H2F2 Constrained at 0.25 Check
PLAT300_ALERT_4_G Atom Site Occupancy of H2F3 Constrained at 0.25 Check
PLAT300_ALERT_4_G Atom Site Occupancy of N1G Constrained at 0.25 Check
PLAT300_ALERT_4_G Atom Site Occupancy of C1G Constrained at 0.25 Check
PLAT300_ALERT_4_G Atom Site Occupancy of C2G Constrained at 0.25 Check
PLAT300_ALERT_4_G Atom Site Occupancy of H1G1 Constrained at 0.25 Check
PLAT300_ALERT_4_G Atom Site Occupancy of H1G2 Constrained at 0.25 Check
PLAT300_ALERT_4_G Atom Site Occupancy of H1G3 Constrained at 0.25 Check
PLAT300_ALERT_4_G Atom Site Occupancy of H1G4 Constrained at 0.25 Check
PLAT300_ALERT_4_G Atom Site Occupancy of H1G5 Constrained at 0.25 Check
PLAT300_ALERT_4_G Atom Site Occupancy of H2G1 Constrained at 0.25 Check
PLAT300_ALERT_4_G Atom Site Occupancy of H2G2 Constrained at 0.25 Check
PLAT300_ALERT_4_G Atom Site Occupancy of H2G3 Constrained at 0.25 Check
PLAT300_ALERT_4_G Atom Site Occupancy of N1I Constrained at 0.25 Check
PLAT300_ALERT_4_G Atom Site Occupancy of C1I Constrained at 0.25 Check
PLAT300_ALERT_4_G Atom Site Occupancy of C2I Constrained at 0.25 Check
PLAT300_ALERT_4_G Atom Site Occupancy of H1I1 Constrained at 0.25 Check
PLAT300_ALERT_4_G Atom Site Occupancy of H1I2 Constrained at 0.25 Check
PLAT300_ALERT_4_G Atom Site Occupancy of H1I3 Constrained at 0.25 Check
PLAT300_ALERT_4_G Atom Site Occupancy of H1I4 Constrained at 0.25 Check
PLAT300_ALERT_4_G Atom Site Occupancy of H1I5 Constrained at 0.25 Check
PLAT300_ALERT_4_G Atom Site Occupancy of H2I1 Constrained at 0.25 Check
PLAT300_ALERT_4_G Atom Site Occupancy of H2I2 Constrained at 0.25 Check
PLAT300_ALERT_4_G Atom Site Occupancy of H2I3 Constrained at 0.25 Check
PLAT300_ALERT_4_G Atom Site Occupancy of N1K Constrained at 0.25 Check
PLAT300_ALERT_4_G Atom Site Occupancy of C1K Constrained at 0.25 Check
PLAT300_ALERT_4_G Atom Site Occupancy of C2K Constrained at 0.25 Check
PLAT300_ALERT_4_G Atom Site Occupancy of H1K1 Constrained at 0.25 Check
PLAT300_ALERT_4_G Atom Site Occupancy of H1K2 Constrained at 0.25 Check
PLAT300_ALERT_4_G Atom Site Occupancy of H1K3 Constrained at 0.25 Check
PLAT300_ALERT_4_G Atom Site Occupancy of H1K4 Constrained at 0.25 Check
PLAT300_ALERT_4_G Atom Site Occupancy of H1K5 Constrained at 0.25 Check
PLAT300_ALERT_4_G Atom Site Occupancy of H2K1 Constrained at 0.25 Check
PLAT300_ALERT_4_G Atom Site Occupancy of H2K2 Constrained at 0.25 Check
PLAT300_ALERT_4_G Atom Site Occupancy of H2K3 Constrained at 0.25 Check
PLAT300_ALERT_4_G Atom Site Occupancy of N1M Constrained at 0.4 Check
PLAT300_ALERT_4_G Atom Site Occupancy of C1M Constrained at 0.4 Check
PLAT300_ALERT_4_G Atom Site Occupancy of C2M Constrained at 0.4 Check
PLAT300_ALERT_4_G Atom Site Occupancy of H1M1 Constrained at 0.4 Check
PLAT300_ALERT_4_G Atom Site Occupancy of H1M2 Constrained at 0.4 Check
PLAT300_ALERT_4_G Atom Site Occupancy of H1M3 Constrained at 0.4 Check
PLAT300_ALERT_4_G Atom Site Occupancy of H1M4 Constrained at 0.4 Check
PLAT300_ALERT_4_G Atom Site Occupancy of H1M5 Constrained at 0.4 Check
PLAT300_ALERT_4_G Atom Site Occupancy of H2M1 Constrained at 0.4 Check
PLAT300_ALERT_4_G Atom Site Occupancy of H2M2 Constrained at 0.4 Check
PLAT300_ALERT_4_G Atom Site Occupancy of H2M3 Constrained at 0.4 Check
PLAT300_ALERT_4_G Atom Site Occupancy of N1O Constrained at 0.35 Check
PLAT300_ALERT_4_G Atom Site Occupancy of C1O Constrained at 0.35 Check
PLAT300_ALERT_4_G Atom Site Occupancy of C2O Constrained at 0.35 Check
PLAT300_ALERT_4_G Atom Site Occupancy of H1O1 Constrained at 0.35 Check

```
PLAT300_ALERT_4_G Atom Site Occupancy of H1O2       Constrained at       0.35 Check
PLAT300_ALERT_4_G Atom Site Occupancy of H1O3       Constrained at       0.35 Check
PLAT300_ALERT_4_G Atom Site Occupancy of H1O4       Constrained at       0.35 Check
PLAT300_ALERT_4_G Atom Site Occupancy of H1O5       Constrained at       0.35 Check
PLAT300_ALERT_4_G Atom Site Occupancy of H2O1       Constrained at       0.35 Check
PLAT300_ALERT_4_G Atom Site Occupancy of H2O2       Constrained at       0.35 Check
PLAT300_ALERT_4_G Atom Site Occupancy of H2O3       Constrained at       0.35 Check
PLAT300_ALERT_4_G Atom Site Occupancy of O4W’       Constrained at        0.5 Check
PLAT300_ALERT_4_G Atom Site Occupancy of H4WC       Constrained at        0.5 Check
PLAT300_ALERT_4_G Atom Site Occupancy of H4WD       Constrained at        0.5 Check
PLAT300_ALERT_4_G Atom Site Occupancy of O2W        Constrained at        0.5 Check
PLAT300_ALERT_4_G Atom Site Occupancy of H2WA       Constrained at        0.5 Check
PLAT300_ALERT_4_G Atom Site Occupancy of H2WB       Constrained at        0.5 Check
PLAT300_ALERT_4_G Atom Site Occupancy of O4W        Constrained at        0.5 Check
PLAT300_ALERT_4_G Atom Site Occupancy of H4WA       Constrained at        0.5 Check
PLAT300_ALERT_4_G Atom Site Occupancy of H4WB       Constrained at        0.5 Check
PLAT300_ALERT_4_G Atom Site Occupancy of O7W        Constrained at        0.5 Check
PLAT300_ALERT_4_G Atom Site Occupancy of H7WA       Constrained at        0.5 Check
PLAT300_ALERT_4_G Atom Site Occupancy of H7WB       Constrained at        0.5 Check
PLAT300_ALERT_4_G Atom Site Occupancy of O5W        Constrained at       0.25 Check
PLAT300_ALERT_4_G Atom Site Occupancy of H5WA       Constrained at       0.25 Check
PLAT300_ALERT_4_G Atom Site Occupancy of H5WB       Constrained at       0.25 Check
PLAT300_ALERT_4_G Atom Site Occupancy of O6W        Constrained at       0.25 Check
PLAT300_ALERT_4_G Atom Site Occupancy of H6WA       Constrained at       0.25 Check
PLAT300_ALERT_4_G Atom Site Occupancy of H6WB       Constrained at       0.25 Check
PLAT300_ALERT_4_G Atom Site Occupancy of O8W        Constrained at       0.25 Check
PLAT300_ALERT_4_G Atom Site Occupancy of O9W        Constrained at       0.25 Check
PLAT300_ALERT_4_G Atom Site Occupancy of H9WA       Constrained at       0.25 Check
PLAT300_ALERT_4_G Atom Site Occupancy of H9WB       Constrained at       0.25 Check
PLAT300_ALERT_4_G Atom Site Occupancy of O10W       Constrained at       0.25 Check
PLAT300_ALERT_4_G Atom Site Occupancy of H10U       Constrained at       0.25 Check
PLAT300_ALERT_4_G Atom Site Occupancy of H10V       Constrained at       0.25 Check
PLAT300_ALERT_4_G Atom Site Occupancy of O12W       Constrained at       0.25 Check
PLAT300_ALERT_4_G Atom Site Occupancy of H12U       Constrained at       0.25 Check
PLAT300_ALERT_4_G Atom Site Occupancy of H12V       Constrained at       0.25 Check
PLAT300_ALERT_4_G Atom Site Occupancy of O13W       Constrained at       0.25 Check
PLAT300_ALERT_4_G Atom Site Occupancy of H13U       Constrained at       0.25 Check
PLAT300_ALERT_4_G Atom Site Occupancy of H13V       Constrained at       0.25 Check
PLAT300_ALERT_4_G Atom Site Occupancy of O14W       Constrained at       0.15 Check
PLAT300_ALERT_4_G Atom Site Occupancy of H14U       Constrained at       0.15 Check
PLAT300_ALERT_4_G Atom Site Occupancy of H14V       Constrained at       0.15 Check
PLAT300_ALERT_4_G Atom Site Occupancy of H8WA       Constrained at       0.25 Check
PLAT300_ALERT_4_G Atom Site Occupancy of H8WB       Constrained at       0.25 Check
PLAT301_ALERT_3_G Main Residue  Disorder .............(Resd  1  )        58% Note
PLAT302_ALERT_4_G Anion/Solvent/Minor-Residue Disorder (Resd  2  )       100% Note
PLAT302_ALERT_4_G Anion/Solvent/Minor-Residue Disorder (Resd  3  )       100% Note
PLAT302_ALERT_4_G Anion/Solvent/Minor-Residue Disorder (Resd  4  )       100% Note
PLAT302_ALERT_4_G Anion/Solvent/Minor-Residue Disorder (Resd  5  )       100% Note
PLAT302_ALERT_4_G Anion/Solvent/Minor-Residue Disorder (Resd  6  )       100% Note
PLAT302_ALERT_4_G Anion/Solvent/Minor-Residue Disorder (Resd  7  )       100% Note
PLAT302_ALERT_4_G Anion/Solvent/Minor-Residue Disorder (Resd  8  )       100% Note
PLAT302_ALERT_4_G Anion/Solvent/Minor-Residue Disorder (Resd  9  )       100% Note
PLAT302_ALERT_4_G Anion/Solvent/Minor-Residue Disorder (Resd 10  )       100% Note
PLAT302_ALERT_4_G Anion/Solvent/Minor-Residue Disorder (Resd 11  )       100% Note
PLAT302_ALERT_4_G Anion/Solvent/Minor-Residue Disorder (Resd 12  )       100% Note
PLAT302_ALERT_4_G Anion/Solvent/Minor-Residue Disorder (Resd 13  )       100% Note
PLAT302_ALERT_4_G Anion/Solvent/Minor-Residue Disorder (Resd 14  )       100% Note
PLAT302_ALERT_4_G Anion/Solvent/Minor-Residue Disorder (Resd 15  )       100% Note
PLAT302_ALERT_4_G Anion/Solvent/Minor-Residue Disorder (Resd 16  )       100% Note
PLAT302_ALERT_4_G Anion/Solvent/Minor-Residue Disorder (Resd 17  )       100% Note
PLAT302_ALERT_4_G Anion/Solvent/Minor-Residue Disorder (Resd 18  )       100% Note
PLAT302_ALERT_4_G Anion/Solvent/Minor-Residue Disorder (Resd 20  )       100% Note
```

```
PLAT302_ALERT_4_G Anion/Solvent/Minor-Residue Disorder (Resd 21  )       100% Note
PLAT302_ALERT_4_G Anion/Solvent/Minor-Residue Disorder (Resd 22  )       100% Note
PLAT302_ALERT_4_G Anion/Solvent/Minor-Residue Disorder (Resd 23  )       100% Note
PLAT302_ALERT_4_G Anion/Solvent/Minor-Residue Disorder (Resd 24  )       100% Note
PLAT302_ALERT_4_G Anion/Solvent/Minor-Residue Disorder (Resd 25  )       100% Note
PLAT302_ALERT_4_G Anion/Solvent/Minor-Residue Disorder (Resd 26  )       100% Note
PLAT302_ALERT_4_G Anion/Solvent/Minor-Residue Disorder (Resd 27  )       100% Note
PLAT311_ALERT_2_G Isolated Disordered Oxygen Atom (No H's ?) .....        O8W Check
PLAT415_ALERT_2_G Short Inter D-H..H-X       H37N     ..H2C5     .       1.64 Ang.
                                                 -x,1-y,1-z  =      2_566 Check
PLAT415_ALERT_2_G Short Inter D-H..H-X       H37N     ..H2C2     .       2.09 Ang.
                                                 -x,1-y,1-z  =      2_566 Check
PLAT415_ALERT_2_G Short Inter D-H..H-X       H11V     ..H1K4     .       1.97 Ang.
                                                      x,y,z  =      1_555 Check
PLAT415_ALERT_2_G Short Inter D-H..H-X       H11V     ..H1K5     .       1.69 Ang.
                                                      x,y,z  =      1_555 Check
PLAT415_ALERT_2_G Short Inter D-H..H-X       H11V     ..H1M4     .       1.64 Ang.
                                                 1-x,1-y,-z  =      2_665 Check
PLAT415_ALERT_2_G Short Inter D-H..H-X       H11V     ..H1M5     .       1.83 Ang.
                                                 1-x,1-y,-z  =      2_665 Check
PLAT417_ALERT_2_G Short Inter D-H..H-D       H37M     ..H4WA     .       1.71 Ang.
                                                      x,y,z  =      1_555 Check
PLAT417_ALERT_2_G Short Inter D-H..H-D       H37N     ..H4WA     .       1.98 Ang.
                                                      x,y,z  =      1_555 Check
PLAT417_ALERT_2_G Short Inter D-H..H-D       H42N     ..H2WA     .       2.07 Ang.
                                                    x,1+y,z  =      1_565 Check
PLAT417_ALERT_2_G Short Inter D-H..H-D       H42N     ..H2WB     .       2.00 Ang.
                                                    x,1+y,z  =      1_565 Check
PLAT417_ALERT_2_G Short Inter D-H..H-D       H42N     ..H7WB     .       2.06 Ang.
                                                    x,1+y,z  =      1_565 Check
PLAT432_ALERT_2_G Short Inter X...Y Contact  O4W'     ..C2C              2.95 Ang.
                                                      x,y,z  =      1_555 Check
PLAT432_ALERT_2_G Short Inter X...Y Contact  O3'      ..C9               2.97 Ang.
                                                  -x,2-y,-z  =      2_575 Check
PLAT432_ALERT_2_G Short Inter X...Y Contact  O4W      ..C2C              2.76 Ang.
                                                      x,y,z  =      1_555 Check
PLAT432_ALERT_2_G Short Inter X...Y Contact  O13'     ..C2               3.02 Ang.
                                                  -x,2-y,-z  =      2_575 Check
PLAT432_ALERT_2_G Short Inter X...Y Contact  O22'     ..C5               2.97 Ang.
                                                 -x,2-y,1-z  =      2_576 Check
PLAT432_ALERT_2_G Short Inter X...Y Contact  O8       ..C13              3.02 Ang.
                                                 -x,2-y,1-z  =      2_576 Check
PLAT432_ALERT_2_G Short Inter X...Y Contact  O13W     ..C19              2.84 Ang.
                                                  -x,1-y,-z  =      2_565 Check
PLAT432_ALERT_2_G Short Inter X...Y Contact  O25      ..C6               3.01 Ang.
                                                   x,-1+y,z  =      1_545 Check
PLAT432_ALERT_2_G Short Inter X...Y Contact  C19      ..C1K              3.01 Ang.
                                                   -1+x,y,z  =      1_455 Check
PLAT432_ALERT_2_G Short Inter X...Y Contact  C19      ..C1M              3.16 Ang.
                                                  -x,1-y,-z  =      2_565 Check
PLAT720_ALERT_4_G Number of Unusual/Non-Standard Labels ..........        139 Note
PLAT773_ALERT_2_G Check long C-C Bond in CIF: C2C      --C2N             1.92 Ang.
PLAT779_ALERT_4_G Suspect or Irrelevant (Bond) Angle(s) in CIF . #        314 Check
              N1C' -C2C  -C2N     1.555   1.555   1.556             43.30 Deg.
PLAT780_ALERT_1_G Coordinates do not Form a Properly Connected Set     Please Do !
PLAT789_ALERT_4_G Atoms with Negative _atom_site_disorder_group  #         55 Check
PLAT811_ALERT_5_G No ADDSYM Analysis: Too Many Excluded Atoms ....          ! Info
PLAT860_ALERT_3_G Number of Least-Squares Restraints .............       3444 Note
PLAT883_ALERT_1_G No Info/Value for _atom_sites_solution_primary .     Please Do !
PLAT910_ALERT_3_G Missing # of FCF Reflection(s) Below Theta(Min).          4 Note
PLAT912_ALERT_4_G Missing # of FCF Reflections Above STh/L=  0.600        940 Note
PLAT933_ALERT_2_G Number of OMIT Records in Embedded .res File ...          8 Note
```

```
PLAT941_ALERT_3_G Average HKL Measurement Multiplicity ...........        2.2 Low
PLAT992_ALERT_5_G Repd & Actual _reflns_number_gt Values Differ by         3 Check
```

```
  0 ALERT level A = Most likely a serious problem - resolve or explain
  1 ALERT level B = A potentially serious problem, consider carefully
 43 ALERT level C = Check. Ensure it is not caused by an omission or oversight
251 ALERT level G = General information/check it is not something unexpected

  3 ALERT type 1 CIF construction/syntax error, inconsistent or missing data
 62 ALERT type 2 Indicator that the structure model may be wrong or deficient
  9 ALERT type 3 Indicator that the structure quality may be low
217 ALERT type 4 Improvement, methodology, query or suggestion
  4 ALERT type 5 Informative message, check
```

It is advisable to attempt to resolve as many as possible of the alerts in all categories. Often the minor alerts point to easily fixed oversights, errors and omissions in your CIF or refinement strategy, so attention to these fine details can be worthwhile. In order to resolve some of the more serious problems it may be necessary to carry out additional measurements or structure refinements. However, the purpose of your study may justify the reported deviations and the more serious of these should normally be commented upon in the discussion or experimental section of a paper or in the "special_details" fields of the CIF. checkCIF was carefully designed to identify outliers and unusual parameters, but every test has its limitations and alerts that are not important in a particular case may appear. Conversely, the absence of alerts does not guarantee there are no aspects of the results needing attention. It is up to the individual to critically assess their own results and, if necessary, seek expert advice.

Publication of your CIF in IUCr journals

A basic structural check has been run on your CIF. These basic checks will be run on all CIFs submitted for publication in IUCr journals (*Acta Crystallographica*, *Journal of Applied Crystallography*, *Journal of Synchrotron Radiation*); however, if you intend to submit to *Acta Crystallographica Section C* or *E* or *IUCrData*, you should make sure that full publication checks are run on the final version of your CIF prior to submission.

Publication of your CIF in other journals

Please refer to the *Notes for Authors* of the relevant journal for any special instructions relating to CIF submission.

PLATON version of 22/04/2020; check.def file version of 09/03/2020

Datablock mo_TrNi13_0m - ellipsoid plot

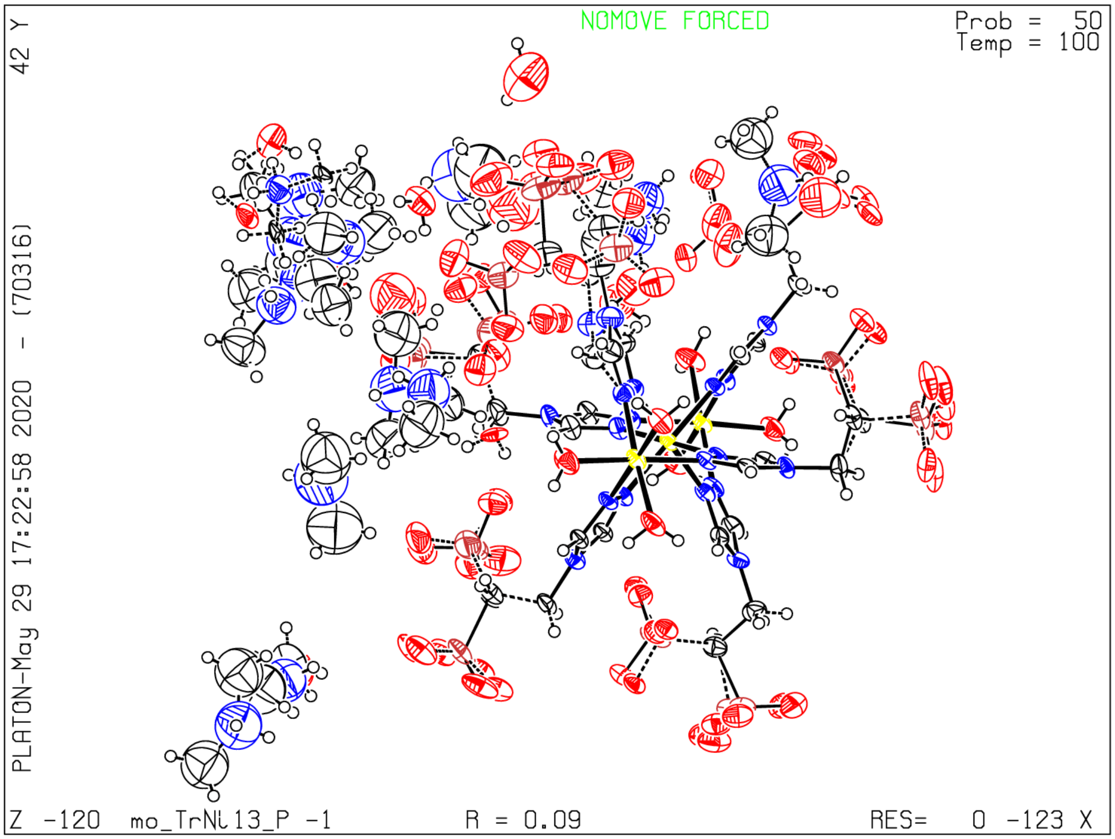

Supplement: Supplementary file 1 [file molecules-26-06020-s001.zip › molecules-1380646-supplementary.pdf]
